# A correctable immune niche for epithelial stem cell reprogramming and post-viral lung diseases

**DOI:** 10.1172/JCI183092

**Published:** 2024-07-25

**Authors:** Kangyun Wu, Yong Zhang, Huiqing Yin-DeClue, Kelly Sun, Dailing Mao, Kuangying Yang, Stephen R. Austin, Erika C. Crouch, Steven L. Brody, Derek E. Byers, Christy M. Hoffmann, Michael E. Hughes, Michael J. Holtzman

**Affiliations:** 1 Pulmonary and Critical Care Medicine, Department of Medicine,; 2 Department of Pathology and Immunology,; 3 Department of Genetics, and; 4 Department of Cell Biology and Physiology, Washington University School of Medicine, St. Louis, Missouri, USA.

**Keywords:** Pulmonology, Adult stem cells, Influenza, Respiration

## Abstract

Epithelial barriers are programmed for defense and repair but are also the site of long-term structural remodeling and disease. In general, this paradigm features epithelial stem cells (ESCs) that are called on to regenerate damaged tissues but can also be reprogrammed for detrimental remodeling. Here we identified a *Wfdc21-*dependent monocyte-derived dendritic cell (moDC) population that functioned as an early sentinel niche for basal ESC reprogramming in mouse models of epithelial injury after respiratory viral infection. Niche function depended on moDC delivery of ligand GPNMB to the basal ESC receptor CD44 so that properly timed antibody blockade of ligand or receptor provided long-lasting correction of reprogramming and broad disease phenotypes. These same control points worked directly in mouse and human basal ESC organoids. Together, the findings identify a mechanism to explain and modify what is otherwise a stereotyped but sometimes detrimental response to epithelial injury.

## Abstract 2

This study defines the immune and epithelial cell network that responds to lung injury and how to prevent the consequence of structural remodeling and disease.

## Graphical abstract

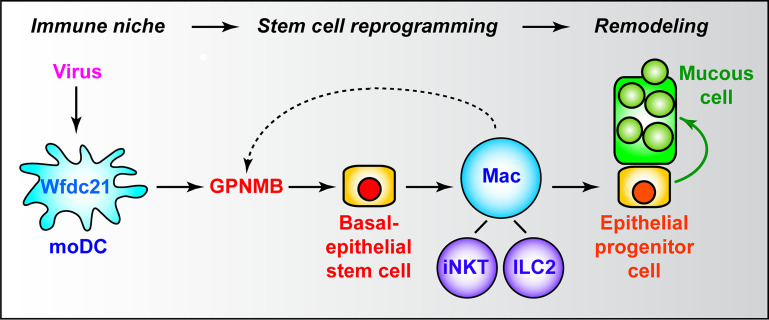

## Introduction

Epithelial barriers maintain a fundamental responsibility to respond to injuries from the full range of environmental toxins and infectious agents. This role depends critically on carefully controlled growth and differentiation of epithelial stem cells (ESCs) and the coordinated actions of resident and recruited immune cells. In optimal circumstances, this epithelial-immune cell program restores proper barrier integrity, but in some cases, ESCs can be reprogrammed for an excessive and prolonged response that results in long-term structural remodeling and disease. Thus, it is critical to define the cell and molecular explanation for ESC reprogramming, particularly as a mechanism that might be amenable to correction and consequent disease modification. In that regard, it is also key to understand how this stem cell checkpoint is interfaced to the immune cell network. At present, however, the full basis for homeostatic versus divergent barrier responses and a corresponding need for a disease modifier still need to be better addressed.

In that context, the respiratory epithelial barrier represents an extremely common site of injury and dysfunction and consequently a compelling practical target for study. Indeed, there is significant evidence already for stem cell function for epithelial basal cells, alveolar type 2 (AT2) cells, and club cells along with hybrid and transitional cell populations (1–15) in orchestrating barrier repair in the lung. Further, there is a well-studied requirement for companion resident and recruited immune cells, particularly lung monocytes and macrophages, in lung tissue repair and regeneration (16–20), following the line of investigation in other organs (20–25). However, as introduced above, the precise cell and molecular connections between ESCs and the immune cell network have not reached a level of understanding to deliver a disease-modifying strategy.

This unmet need is particularly underscored in the case of common and sometimes pandemic conditions for respiratory viral infection and the high percentage of respiratory viral infections turning into post-viral lung disease (PVLD). As a starting point for mechanism, this type of injury uniformly activates basal ESCs in experimental models and clinical conditions across the spectrum of established and emergent viruses (26). Further, the initial infectious illness commonly progresses to structural remodeling that features basal ESC reprogramming and consequently impaired lung function (27). This pattern is preserved across established pathogens like influenza A virus (IAV) and emerging agents like SARS-CoV-2 wherein acute infection transitions to chronic disease in experimental and clinical conditions (11, 12, 28). Other common viruses, like respiratory syncytial virus, can initiate severe infections in infancy that (alone or in synergy with other inhaled triggers) result in lifelong lung disease phenotypes in the form of asthma and chronic obstructive pulmonary disease (29–34). In all cases, the basal ESC interface to immune cell function also needs to be better defined (35). In particular, the selective cues for basal ESC and immune cell overactivation still need to be determined as a biologic, immunologic, and therapeutic endpoint.

Here we address these endpoints with matching experimental approaches that are then subject to validation in initial but comparable human studies. This style serves as the basis for the present study. Thus, we engage a mouse model with a native viral pathogen that preserved the natural pattern of proximal to distal spread of infection and the basal ESC reprogramming that drives structural remodeling and PVLD with pulmonary dysfunction (27, 36–41). In turn, the features of this model accurately predicted corresponding biomarkers in clinical samples from humans with chronic lung disease linked to viral infection (28, 38, 42, 43). Consequently, in the present work, this model served as the starting point for cell-specific and single-cell analysis of gene expression that identified *Wfdc21* and *Gpnmb* gene products for functional study. Historically, these gene products were connected to monocyte-derived dendritic cell (moDC) differentiation (44) and tumor infiltration (45–47) but were not combined under those conditions or related in any way to post-viral remodeling and disease. Unexpectedly, the present functional analysis revealed the early post-viral appearance of a sentinel population of *Wfdc21*-dependent moDCs as an immune niche for basal ESC reprograming, and identified the niche-derived signal as glycoprotein nonmetastatic melanoma B (GPNMB; also called osteoactivin) and the functional basal ESC receptor for GPNMB as CD44, also not previously linked to the PVLD process. Moreover, this signaling pathway is preserved across native animal and adapted human viruses consistent with a stereotyped response to epithelial barrier injury. The nature of the mechanism translates to blockade of GPNMB ligand or its receptor, which in turn corrects the full spectrum of disease phenotypes (basal cell hyperplasia, metaplasia, immune activation, and mucinous differentiation). Further, the findings were again validated in human cell models of basal ESC growth. The findings thereby provide a fundamental guideline for ESC reprogramming at otherwise protective barrier sites and a basis for broad-based disease modification.

## Results

### Wfdc21^+^ moDCs are required for basal ESC reprogramming and PVLD.

**Figure 1:**
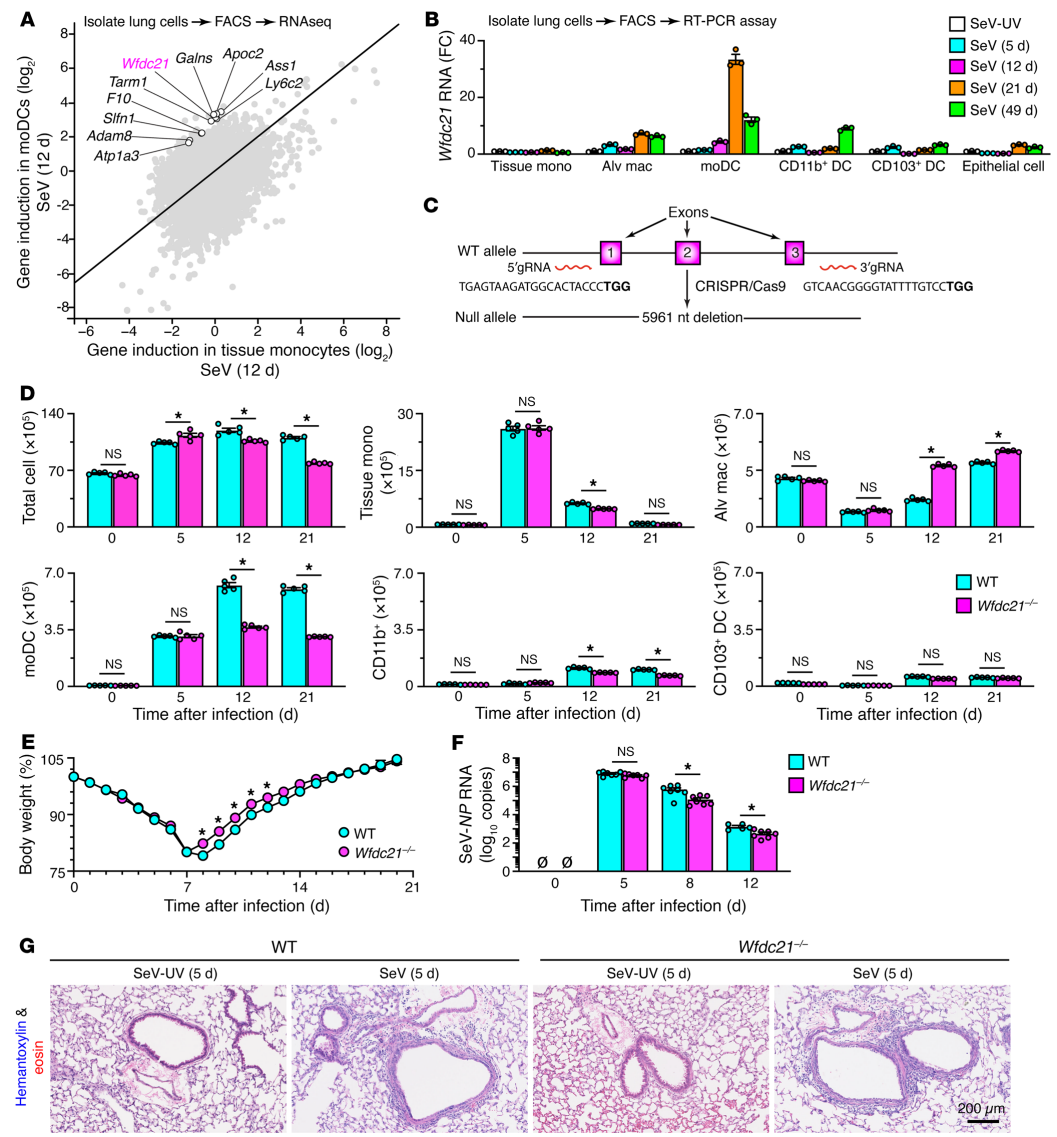
Effect of Wfdc21 on the development of the lung moDC niche. (**A**) RNA-Seq analysis of induction of gene expression in lung tissue monocytes versus moDCs at 12 days after infection with SeV. Annotations indicate 10 genes with the greatest differences in expression level. (**B**) Levels of *Wfdc21* mRNA in immune and epithelial cell populations from FACS of lung tissue at 5–49 days after SeV infection or SeV-UV control. (**C**) Scheme for CRISPR/Cas9–based deletion of *Wfdc21* gene in mice. (**D**) Flow cytometry–derived numbers of immune cells in lung tissue from wild-type (WT) and *Wfdc21^–/–^* mice at 0–21 days after SeV infection. (**E**) Body weights of WT and *Wfdc21^–/–^* mice at 0–21 days after SeV infection. (**F**) SeV-*NP* RNA levels in lung tissue from WT and *Wfdc21^–/–^* mice at 0–12 days after SeV infection. (**G**) Hematoxylin and eosin staining of lung sections from WT and *Wfdc21^–/–^* mice at 5 days after SeV or SeV-UV. Scale bar: 200 μm. Values represent mean ± SEM (*n* = 5–7 mice per condition). *P < 0.05 by ANOVA and Tukey’s correction.

Based on earlier work, we reasoned that moDCs might be specifically required for ESC growth in mouse models of PVLD, particularly after infection with native Sendai virus (SeV), which targets airway epithelial barrier cells (27, 36, 37, 48). Accordingly, we isolated lung immune cell populations with FACS based on a scheme described previously (41, 49) (Supplemental Figure 1; supplemental material available online with this article; https://doi.org/10.1172/JCI183092DS1) and then examined the gene expression profile for moDCs versus tissue monocytes obtained at 12 days after SeV infection when markers that reflect basal ESC growth (reflecting increases in cell size and numbers) are maximal (27). The comparative analysis of gene expression identified *Wfdc21* among the most differentially expressed genes in moDCs (Figure 1A), consistent with a requirement for long noncoding RNA *Wfdc21* for differentiation of human moDCs studied ex vivo (44). Similarly, *Wfdc21* expression was localized primarily to moDCs in mice in vivo based on analysis of lung immune cell subsets and total lung epithelial cells obtained by FACS (27, 41, 49) after SeV infection, particularly at 12 and 21 days after infection (Figure 1B). To assess *Wfdc21* function in vivo, we generated *Wfdc21* gene–knockout mice (*Wfdc21^–/–^*) using CRISPR/Cas9–mediated editing (Figure 1C). These mice showed significant and relatively selective attenuation of the usual increase in moDCs in the lung at 12 and 21 days after SeV infection (Figure 1D). Despite this effect, the acute infectious illness (as tracked by weight loss, viral clearance, and lung histology) was little changed at 5–12 days after SeV infection (Figure 1, E–G).

**Figure 2:**
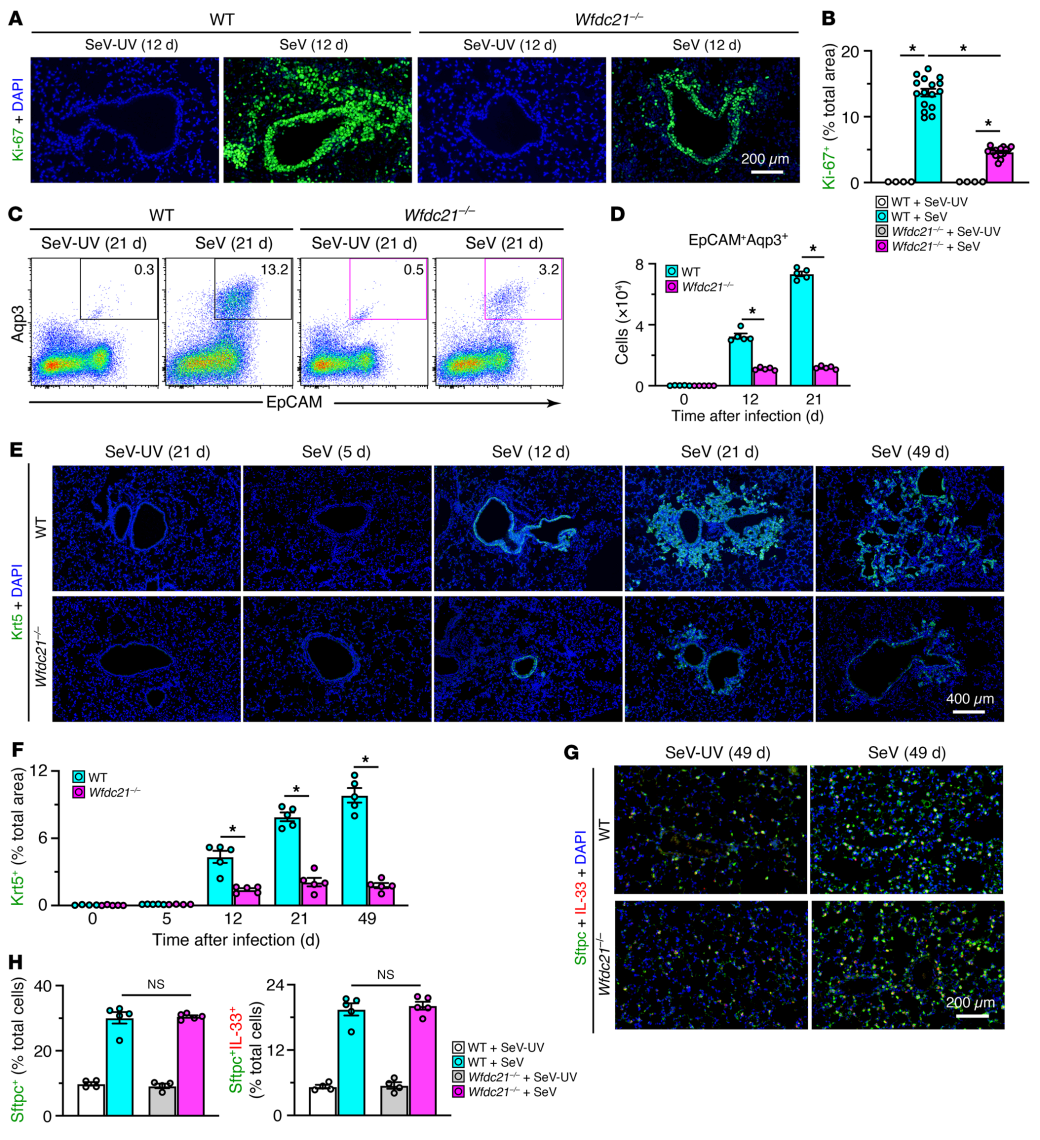
Effect of the Wfdc21-dependent lung moDC niche on basal ESC expansion. (**A**) Immunostaining for Ki-67 in lung sections from WT and *Wfdc21^–/–^* mice at 12 days after SeV infection or SeV-UV control. Scale bar: 200 μm. (**B**) Quantitation of staining in **A**. (**C**) Flow cytograms of lung epithelial (CD31^–^CD45^–^EpCAM^+^Aqp3^+^) cells from WT and *Wfdc21^–/–^* mice at 21 days after SeV or SeV-UV. (**D**) Flow cytometry–derived numbers of cells for conditions in **C**. (**E**) Immunostaining for Krt5 in lung sections from WT and *Wfdc21^–/–^* mice at 0–49 days after SeV or SeV-UV. Scale bar: 400 μm. (**F**) Quantitation of staining in **E**. (**G**) Immunostaining for Sftpc and IL-33 in WT and *Wfdc21^–/–^* mice at 49 days after SeV or SeV-UV. Scale bar: 200 μm. (**H**) Quantitation of staining in **G**. Values represent mean ± SEM (*n* = 4–5 mice per condition). *P < 0.05 by ANOVA and Tukey’s correction.

In contrast to similar acute illness, we found marked attenuation of the usual progression to excess basal cell growth after viral infection in *Wfdc21^–/–^* mice. Thus, increases in the basal ESC proliferation pool based on Ki-67^+^ immunostaining in lung tissue (Figure 2, A and B) and basal epithelial cell levels tracked by flow cytometry (Figure 2, C and D) and Krt5^+^ immunostaining in lung tissue (Figure 2, E and F) were all downregulated in post-viral *Wfdc21^–/–^* mice. In contrast, the increased levels of AT2 cells monitored with Sftpc^+^ and Sftpc^+^IL-33^+^ immunostaining in lung tissue were not significantly different in post-viral *Wfdc21^–/–^* compared with wild-type (WT) control mice (Figure 2, G and H). This finding contrasts with attenuation of AT2 cell growth in *Tlr3^–/–^* mice (49), suggesting a distinct role for *Wfdc21* in selective control of excess basal ESC growth after viral infection.

**Figure 3:**
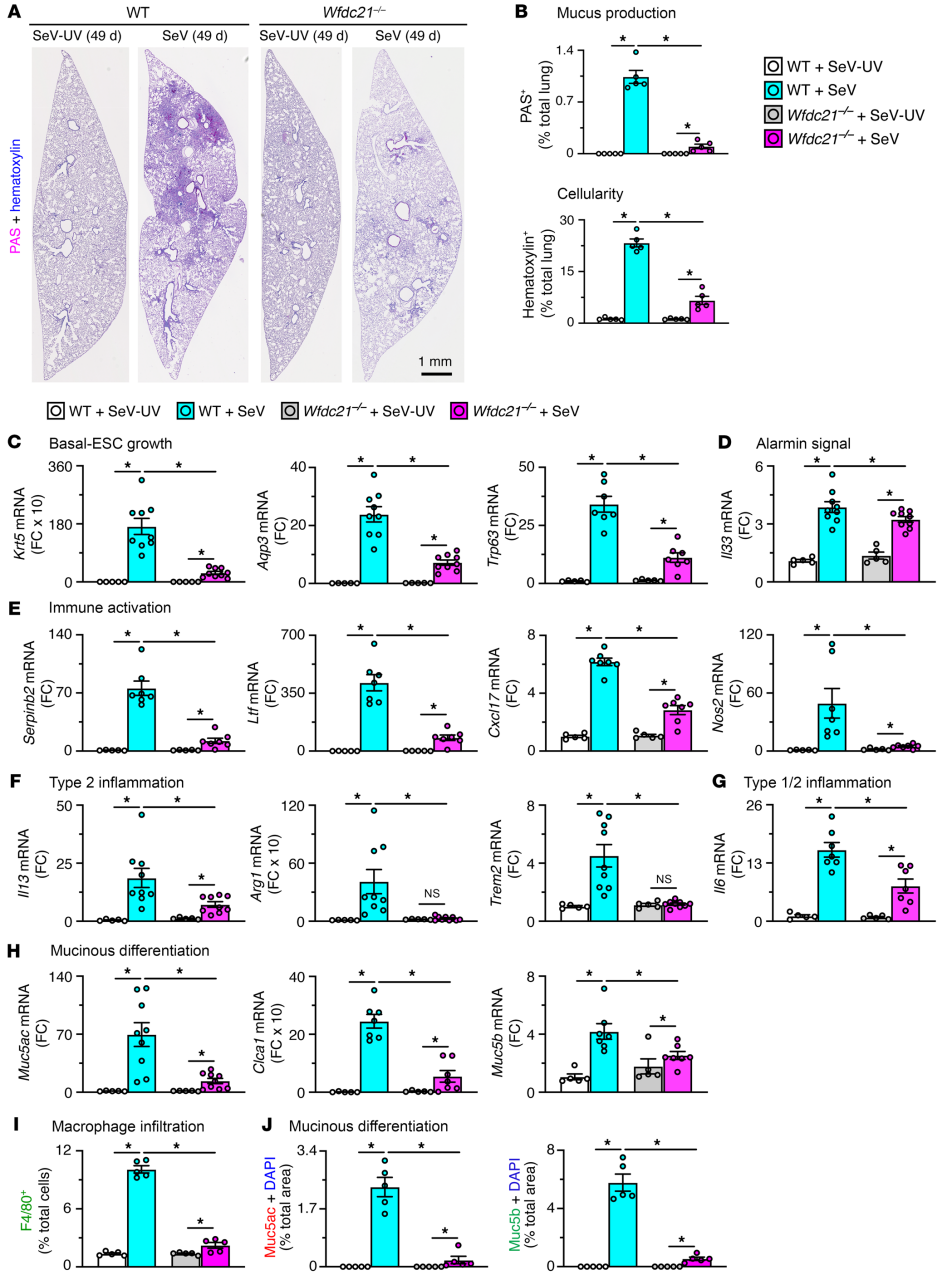
Effect of the Wfdc21-dependent moDC niche on PVLD. (**A**) PAS and hematoxylin staining of lung sections from WT and *Wfdc21^–/–^* mice at 49 days after SeV infection or SeV-UV. Scale bar: 1 mm. (**B**) Quantitation of staining in **A**. (**C**–**H**) Lung tissue levels of mRNA biomarkers in WT and *Wfdc21^–/–^* mice at 49 days after SeV or SeV-UV to track basal ESC growth (**C**), alarmin signal (**D**), immune activation (**E**), type 2 inflammation (**F**), type 1/2 inflammation (**G**), and mucinous differentiation (**H**). (**I**) Quantitation of immunostaining for F4/80 with DAPI counterstaining in lung sections for conditions in **A**. (**J**) Quantitation of immunostaining for Muc5ac and Muc5b with DAPI counterstaining in lung sections for conditions in **A**. Values represent mean ± SEM (*n* = 5–10 mice per condition). *P < 0.05 by ANOVA and Tukey’s correction.

The observed control of basal ESC growth translated to decreases in lung histopathology. In particular, periodic acid–Schiff (PAS) and hematoxylin tissue staining as a reflection of mucus production and cellularity, respectively, was decreased in lung sections at 49 days after SeV infection when PVLD is otherwise maximal (Figure 3, A and B). We also detected significant changes in biomarkers designed to track the post-viral disease pathway. Thus, we found decreases in basal cell growth marked by *Krt5* and *Aqp3* mRNA, alarmin signal marked by *Il33* mRNA, type 2 inflammation marked by *Il13*, *Arg1*, *Trem2*, and *Il6* mRNA, and mucinous differentiation marked by *Muc5ac*, *Clca1*, and *Muc5b* mRNA in *Wfdc21^–/–^* mice (Figure 3, C–H). The decreased *Il33* signal was consistent with blockade of IL-33 induction/activation in basal ESC growth as reported previously (27). These effects were accompanied by decreases in macrophage infiltration based on F4/80^+^ immunostaining (Figure 3I and Supplemental Figure 2A), and mucinous differentiation based on Mucin-5ac (Muc5ac) and Mucin-5b (Muc5b) immunostaining (Figure 3J and Supplemental Figure 2B). The combined findings link *Wfdc21* function to moDC niche support of basal ESC reprogramming and, in turn, downstream type 2 inflammation and mucus production typical of PVLD modeled with SeV infection and relevant to clinical disease (28, 33, 34, 38, 42, 43). To further determine *Wfdc21* control with a human pathogen, we also studied the response to influenza A virus (IAV), which similarly triggers basal ESC growth and PVLD in mouse models and long-term PVLD in humans (11, 12, 39, 50). In this case, *Wfdc21^–/–^* mice also showed no significant change in acute infectious illness or viral clearance (Supplemental Figure 3, A and B) and demonstrated marked attenuation of basal cell hyperplasia, mucus production, and hypercellularity compared with WT mice after infection with IAV (Supplemental Figure 3, C–F), using the same PR8 strain as studied previously (11, 12). The result provided evidence that *Wfdc21^+^* moDC niche control was preserved across different types of viral infections.

### Gpnmb is required for moDC niche function in PVLD.

**Figure 4:**
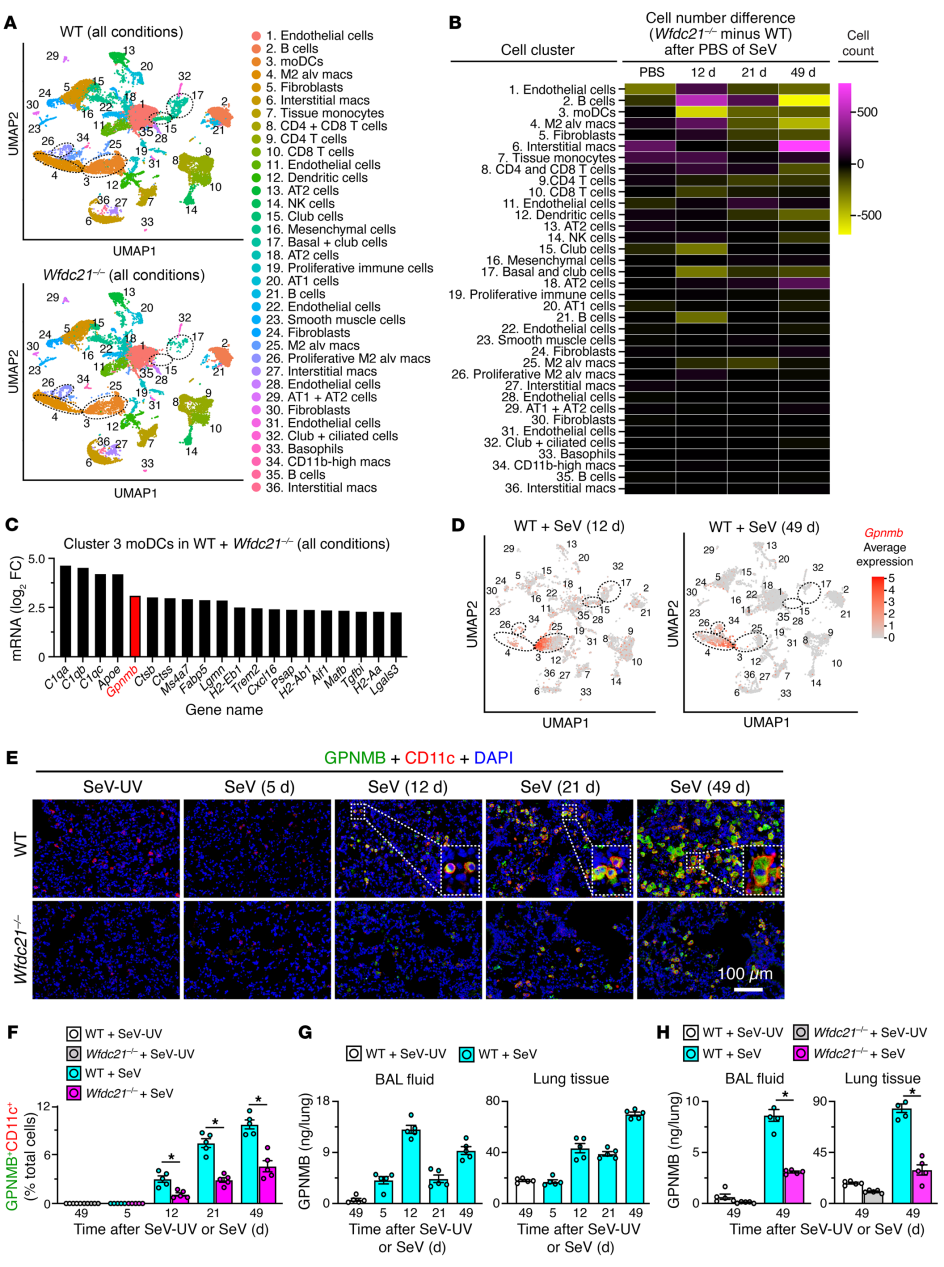
Identification of *Gpnmb* gene expression in moDCs. (**A**) Gene cluster assignments for WT and *Wfdc21^–/–^* mice for all sample conditions. (**B**) Differences in numbers of cells per cluster for WT versus *Wfdc21^–/–^* mice. (**C**) Most highly expressed genes in cluster 3 (moDCs) relative to other clusters. Red annotation denotes *Gpnmb* mRNA expression. (**D**) *Gpnmb* gene expression by cluster analysis for annotations in **A** with the highest signals in moDCs (cluster 3) at 12 days and M2-alveolar macrophages (cluster 4) at 49 days. (**E**) Immunostaining for GPNMB and CD11c with DAPI counterstaining in lung sections from WT and *Wfdc21^–/–^* mice at 5–49 days after SeV and 49 days after SeV-UV. Scale bar: 100 μm. Insets, magnification ×6. (**F**) Quantitation of staining in **E**. (**G**) Levels of GPNMB in bronchoalveolar lavage (BAL) fluid and lung tissue at 5–49 days after SeV or SeV-UV in WT mice. (**H**) Levels of GPNMB at 49 days after SeV or SeV-UV in WT and *Wfdc21^–/–^* mice (WT + SeV-UV values from **G** data). Values represent mean ± SEM (*n* = 5 mice per condition). *P < 0.05 by ANOVA and Tukey’s correction.

To better define *Wfdc21* and moDC function in PVLD, we next compared gene expression in WT versus *Wfdc21^–/–^* mice using single-cell RNA sequencing (scRNA-Seq) of whole-lung samples from mice after SeV infection. Gene expression patterns across sample conditions (12, 21, and 49 days after SeV infection and PBS control) identified 36 clusters of lung cells based on nearest neighbor analysis (Figure 4A). Comparison of WT with *Wfdc21^–/–^* mice revealed marked decreases in moDCs (cluster 3) along with basal cells (cluster 17) and basal-lineage club cells (clusters 15 and 17) in *Wfdc21^–/–^* mice compared with WT controls (Figure 4A). Quantitative comparisons of each cluster for each condition confirmed this finding and localized the decrease in moDCs to 12 days after SeV infection (Figure 4B). Cluster analysis and cell cycle gene expression mapped onto cell-type clusters at 12 days after infection also showed appearance of cycling basal ESCs in WT mice consistent with our previous analysis (27) and near absence of these cells in *Wfdc21^–/–^* mice at 12 days after SeV infection (Supplemental Figure 4, A and B). To define the moDC signal for basal ESC growth, a review of genes expressed in moDCs revealed prominent expression of *Gpnmb* (Figure 4C) as a candidate that could increase stemness in cancer cells (47). Indeed, gene feature plots showed *Gpnmb* mRNA expression localized to moDCs (cluster 3) at 12 days after SeV infection that shifted to M2-alveolar macrophages by 49 days after SeV infection (Figure 4D), a shift that correlated with induction of *Gpnmb* mRNA in lung tissue (Supplemental Figure 4C). In addition, the appearance of *Gpnmb* in lung tissue coincided with immune activation of basal and basal-lineage cells marked by expression of the chemokine *Cxcl17* (Supplemental Figure 4, C–E). Similarly, GPNMB protein was colocalized in lung tissue to CD11c^+^ cells with primarily moDC morphology at early time points (12 days) and then also alveolar macrophage appearance at late time points (49 days) after infection (Figure 4, E and F). All of these signals were markedly attenuated in *Wfdc21^–/–^* compared with WT mice (Figure 4, E and F). Levels of GPNMB in bronchoalveolar lavage (BAL) fluid and lung tissue showed an early peak at 12 days (particularly in the BAL-detectable secreted form) and a late peak at 49 days (Figure 4G). These findings indicate that immunostaining for GPNMB (that reflects the membrane form) underestimates production, particularly the early contribution from moDCs. Both forms of GPNMB were decreased in *Wfdc21^–/–^* mice (Figure 4H), further supporting moDC production. The same patterns of appearance of *Wfdc21*-dependent *Gpnmb* mRNA expression and GPNMB^+^ CD11c^+^ cells with moDC and macrophage morphology were found after IAV infection (Supplemental Figure 5, A–C), further supporting the same type of response across viral types.

**Figure 5:**
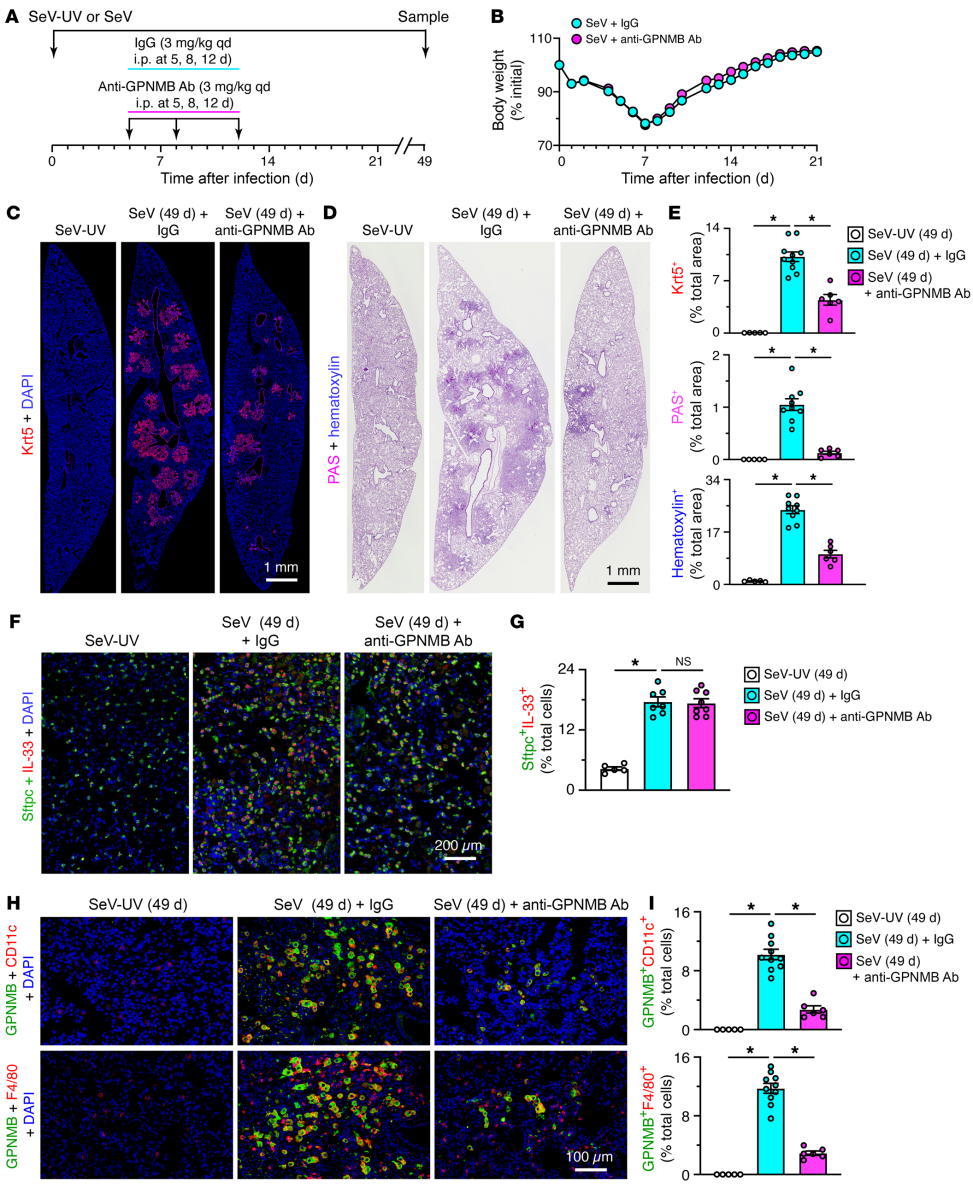
Effect of anti-GPNMB Ab blockade on basal ESC growth and PVLD. (**A**) Protocol scheme for anti-GPNMB Ab or control IgG treatment in mice with SeV infection or SeV-UV control. (**B**) Body weights for conditions in **A**. (**C**) Immunostaining for Krt5 with DAPI counterstaining in lung sections from conditions in **A**. Scale bar: 1 mm. (**D**) PAS and hematoxylin staining of lung sections for conditions in **C**. Scale bar: 1 mm. (**E**) Quantitation of staining in **C** and **D**. (**F**) Immunostaining for Sftpc and IL-33 with DAPI counterstaining in lung sections for conditions in **A**. Scale bar: 200 μm. (**G**) Quantitation of staining in **F**. (**H**) Immunostaining for GPNMB plus CD11c and GPNMB plus F4/80 with DAPI counterstaining for conditions in **A**. Scale bar: 100 μm. (**I**) Quantitation of staining in **H**. Values represent mean ± SEM (*n* = 5–10 mice per condition). *P < 0.05 by ANOVA and Tukey’s correction.

**Figure 6:**
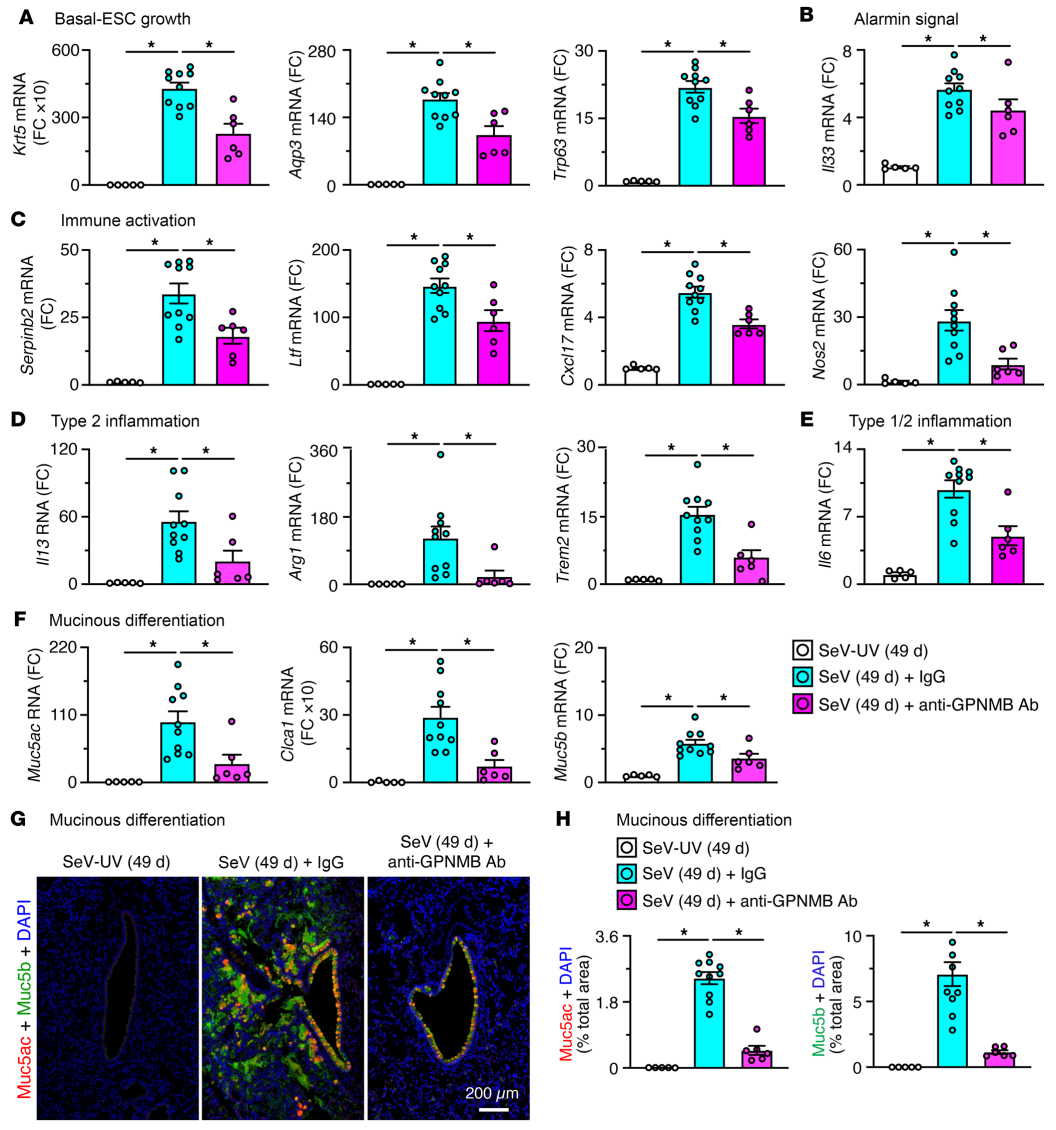
Effect of anti-GPNMB Ab blockade on biomarkers for PVLD. For the protocol scheme for anti-GPNMB Ab or control IgG treatment of mice shown in Figure 5A: (**A**–**F**) Lung tissue levels of mRNA biomarkers in mice at 49 days after SeV infection or SeV-UV to track basal ESC growth (**A**), alarmin signal (**B**), immune activation (**C**), type 2 inflammation (**D**), type 1/2 inflammation (**E**), and mucinous differentiation (**F**). (**G**) Immunostaining for Muc5ac and Muc5b with DAPI counterstaining in lung sections for conditions in **A**. Scale bar: 200 μm. (**H**) Quantitation of staining in **G**. Values represent mean ± SEM (*n* = 5–10 mice per condition). *P < 0.05 by ANOVA and Tukey’s correction.

To define potential function for GPNMB, we determined the effect of anti-GPNMB antibody (Ab) on basal ESC reprogramming and consequent PVLD in the SeV mouse model. In these experiments, anti-GPNMB Ab was dosed at 5, 8, and 12 days after SeV infection (Figure 5A) to target the period of maximal basal ESC proliferation and moDC accumulation the lung. This treatment protocol had no effect on acute illness as marked by body weight loss (Figure 5B). In contrast, anti-GPNMB Ab treatment markedly attenuated basal ESC growth based on Krt5^+^ immunostaining and histopathology assessed with PAS^+^ and hematoxylin^+^ staining of lung tissue at 49 days after SeV infection (Figure 5, C–E). GPNMB blockade had no significant effect on AT2 cell growth or IL-33 expression (Figure 5, F and G) but markedly decreased GPNMB^+^CD11c^+^ moDC-macrophage and GPNMB^+^F4/80^+^ macrophage infiltration at 49 days after infection (Figure 5, H and I). Similarly, treatment decreased basal ESC growth marked by *Krt5*, *Aqp3*, and *Trp63* mRNA, alarmin signal marked by *Il33* mRNA, immune activation marked by *Serpinb2*, *Ltf*, *Cxcl17*, and *Nos2* mRNA, type 2 inflammation marked by *Il13*, *Arg1*, *Trem2*, *and Il6* mRNA, and mucinous differentiation marked by *Muc5ac*, *Clca1*, and *Muc5b* mRNA (Figure 6, A–F), and attenuated mucinous differentiation tracked with Muc5ac and Muc5b immunostaining (Figure 6G) at 49 days after infection. Quantitation of mucin protein staining (Figure 6H) confirmed significant attenuation of these increased signals with anti-GPNMB Ab blockade. The combined findings for GPNMB blockade were all similar to loss of *Wfdc21* function, thereby further linking immune niche–derived GPNMB to control of basal ESC reprogramming and, in turn, downstream type 2 inflammation and mucus production typical of experimental PVLD after infection with SeV or human respiratory enterovirus D68 (27, 40).

### GPNMB/CD44 signaling in basal ESCs drives cell growth and immune activation.

To next determine the GPNMB signaling pathway in basal ESC reprogramming, we analyzed the scRNA-Seq data set with the CellChat package, which integrates gene expression with ligand and receptor signaling databases. The analysis was set to detect significant interactions between basal cells and moDCs versus interactions between basal cells and tissue monocytes (which lack GPNMB expression), and it selected GPNMB-CD44 interaction as having maximal probability for cell-cell communication (Supplemental Figure 6A). As validation, we identified upregulation of KRT5^+^CD44^+^ basal epithelial cells particularly in remodeling regions at 12 days after SeV infection (Supplemental Figure 6B).

**Figure 7:**
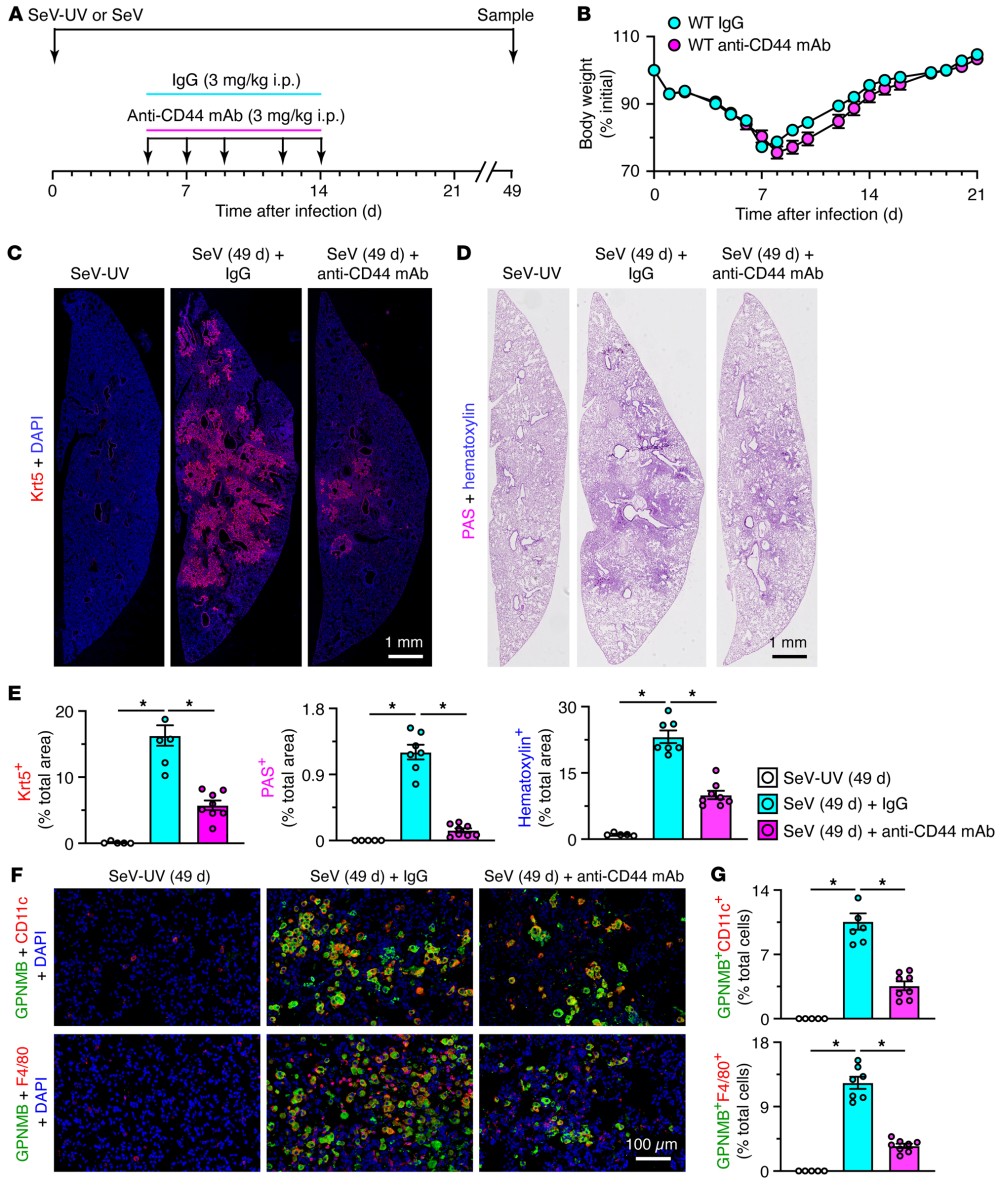
Effect of anti-CD44 Ab blockade on basal ESC reprogramming and PVLD. (**A**) Protocol scheme for anti-CD44 mAb or control IgG treatment in mice with SeV infection compared with SeV-UV control. (**B**) Body weights for conditions in **A**. (**C**) Immunostaining for Krt5 with DAPI counterstaining in lung sections from conditions in **A**. Scale bar: 1 mm. (**D**) PAS and hematoxylin staining of lung sections for conditions in **C**. Scale bar: 1 mm. (**E**) Quantitation of staining in **C** and **D**. (**F**) Immunostaining for GPNMB plus CD11c and GPNMB plus F4/80 with DAPI counterstaining for conditions in **A**. Scale bar: 100 μm. (**G**) Quantitation of staining in **F**. Values represent mean ± SEM (*n* = 5–8 mice per condition). *P < 0.05 by ANOVA and Tukey’s correction.

As done for GPNMB, we determined CD44 function in vivo using anti-CD44 mAb treatment in the SeV mouse model with dosing at 5–14 days after infection (Figure 7A). Here again, treatment did not significantly affect acute illness monitored with body weight loss (Figure 7B) but markedly attenuated basal ESC growth based on KRT5^+^ immunostaining and histopathology tracked with PAS^+^ and hematoxylin^+^ staining of lung tissue at 49 days after SeV infection (Figure 7, C–E). CD44 blockade also markedly decreased GPNMB^+^CD11c^+^ and F4/80^+^ macrophage infiltration at 49 days after infection (Figure 7, F and G). We also observed decreased basal ESC growth, alarmin signal, immune activation, type 2 inflammation, and mucinous differentiation based on mRNA biomarkers (Supplemental Figure 7, A–F) along with decreased mucinous differentiation marked by immunostaining (Supplemental Figure 7, G and H) at 49 days after infection. The combined phenotype for CD44 blockade was similar to *Wfdc21* deficiency and GPNMB blockade, thereby further connecting moDC–basal ESC interaction via GPNMB/CD44 signaling for control of basal ESC reprogramming and consequent PVLD. These findings fit with reports of GPNMB/CD44 signaling in mesenchymal, neural, and cancer cells (51–53).

### GPNMB/CD44 signaling directly controls mouse and human basal ESCs.

**Figure 8:**
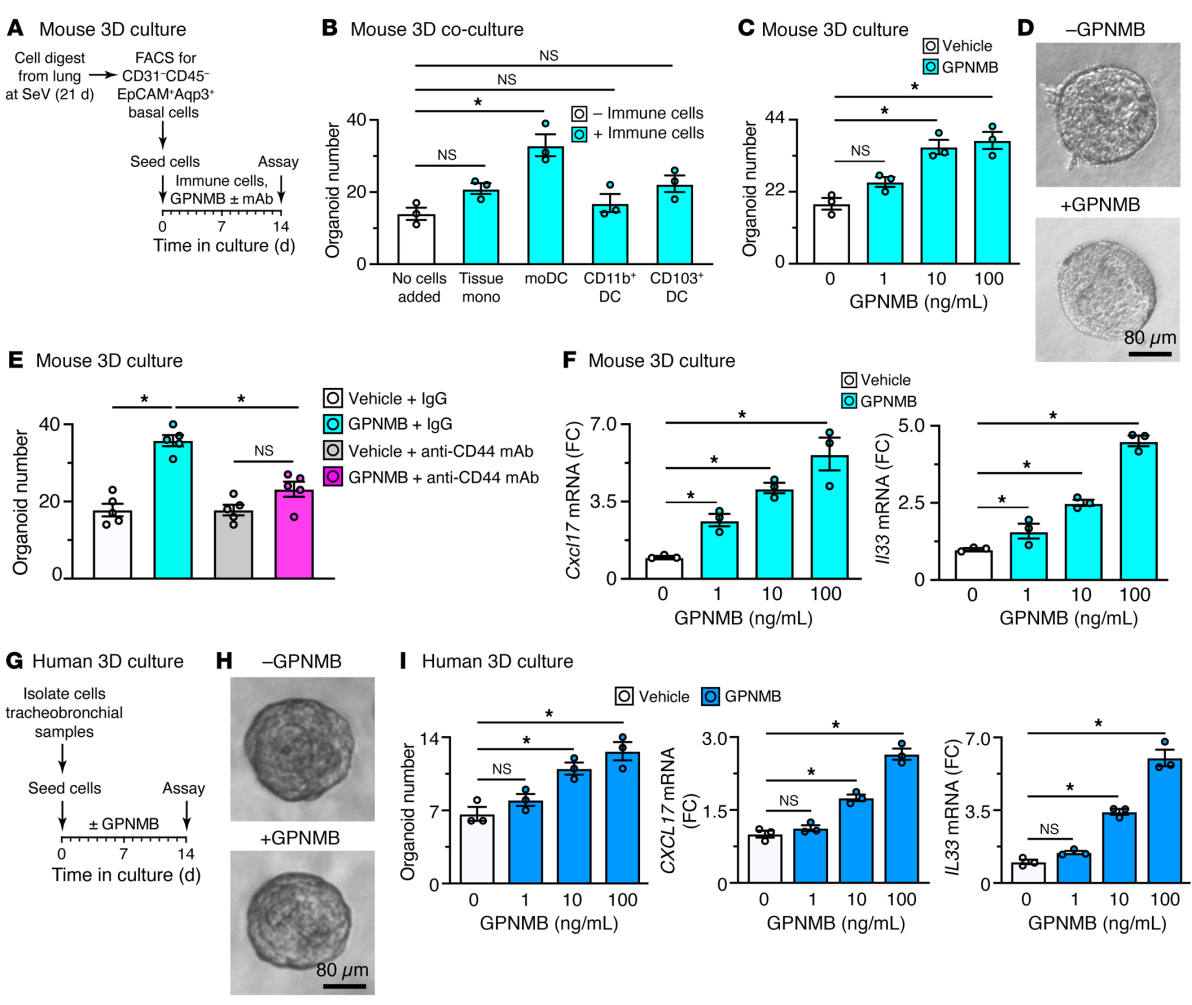
Effect of GPNMB on basal ESC growth and immune activation. (**A**) Protocol scheme for cell isolation from mouse lungs at 21 days after SeV infection, then FACS for CD31^–^CD45^–^EpCAM^+^Aqp3^+^ epithelial cells and seeding into 3D organoid culture. (**B**) Levels of organoid formation from conditions in **A** and coculture with the indicated immune cell populations. (**C**) Levels of organoid formation in 3D cultures incubated with GPNMB (0–100 ng/mL) for conditions in **A**. (**D**) Photomicrographs of organoids for conditions in **C** without or with GPNMB (100 ng/mL). Scale bar: 80 μm. (**E**) Organoid formation for conditions in **C** but using incubation with GPNMB (10 ng/mL) with or without anti-CD44 mAb or control IgG. (**F**) Levels of *Cxcl17* and *Il33* mRNA for conditions in **C**. (**G**) Protocol scheme for human tracheobronchial epithelial cell isolation and 3D culture. (**H**) Photomicrographs of organoids for conditions in **G** without or with GPNMB (100 ng/mL). Scale bar: 80 μm. (**I**) Levels of organoid formation and *CXCL17* and *IL33* mRNA for conditions in **G** with GPNMB (0–100 ng/mL). For **B**–**E** and **G**, results are representative of non-disease control individuals (*n* = 6–8 per condition). *P < 0.05 by ANOVA and Tukey’s correction.

To further determine whether GPNMB/CD44 signal transduction took place directly on basal ESCs, we also assessed function in 3D organoid culture designed for stem cell growth and differentiation (27, 43). Initial experiments showed that basal ESCs isolated from SeV-infected mice and then cocultured with moDCs (but not with tissue macrophages, CD11b^+^ DCs, or CD103^+^ DCs) resulted in more efficient organoid formation as a signature of increased stem cell growth (Figure 8, A and B). Increased organoid formation with characteristic morphology (27) was also found after incubation with recombinant GPNMB (Figure 8, C and D), and this effect was blocked with anti-CD44 mAb (Figure 8E). In addition, we found that addition of GPNMB to 3D organoid cultures also increased expression of *Cxcl17* and *Il33* mRNA (Figure 8F), markers of immune activation for basal and basal-lineage cells as found previously (27, 28, 43) and in the present scRNA-Seq analysis and immunostaining results (Supplemental Figure 4, C–E).

To determine whether the findings in mouse models translate to humans, we assessed GPNMB function in primary 3D organoid cultures using human tracheobronchial cells with the same treatment protocol (Figure 8G). Here again, we found that addition of GPNMB to cultures caused significant increases in basal ESC growth marked by more efficient formation of organoids with typical morphology (43) and immune activation marked by *CXCL17* and *IL33* mRNA (Figure 8, H and I). These findings thereby confirmed activation of a comparable cell and molecular pathway in experimental conditions for lung remodeling diseases linked to respiratory viral infection and perhaps other epithelial barrier injuries.

## Discussion

In this study, we identify and correct an immune cell niche for basal ESC reprogramming and PVLD using mouse models and comparable human cell models and clinical samples. Key findings include (a) *Wfdc21* gene function for moDC development as a niche for basal ESC growth based on mouse models of PVLD; (b) corresponding moDC production of GPNMB as a correctable driver for basal ESC growth and immune activation; (c) linked CD44 signaling on basal ESCs to orchestrate GPNMB function; and (d) comparable GPNMB signaling for mouse and human basal ESC growth and immune activation in 3D organoid cultures. Together, the results provide a general model for PVLD and related diseases (as diagrammed in Supplemental Figure 8). Here we place these findings in context to present the impact for pathogenesis and treatment of PVLD and implications for similar disease conditions at epithelial injury sites.

First, in relation to *Wfdc21* function, the present work provides a precise mechanism for moDC development and differentiation. Previous work showed that *Wfdc21* function depended on its action as a long noncoding RNA that controls STAT3 activation in human cells and mice (44). However, this work was based on siRNA knockdown that might cause off-target effects (54–56). Further, *Wfdc21* encodes a protein (Wdmd1-like) in nearly all species except humans (54–56). The present data for target-specific gene knockout remain consistent with *Wfdc21* RNA function in moDCs and provide evidence of *Wfdc21*-dependent moDCs as a relatively early and well-coordinated immune cell niche for basal ESC expansion. The present analysis of myeloid subsets during inflammation in vivo also demonstrates a later increase in *Wfdc21* expression in alveolar macrophages, in contrast to the previous analysis limited to blood cells (44). The present findings are also consistent with macrophage participation given the time course of full disease development and the downstream effector function of macrophages in PVLD (27, 28, 36–38, 41–43, 57). Notably, *Wfdc21* control of basal ESC growth is preserved in native rodent (SeV) and adapted human (IAV) infection, providing evidence of a stereotyped response to injury that might diverge downstream for distinct disease phenotypes.

Second, in regard to GPNMB function, moDCs were found to be the primary cell site for *Gpnmb* mRNA and corresponding protein induction coinciding with the timing for basal ESC growth. These findings fit with GPNMB discovered for its capacity to regulate DC infiltration along with cancer cell stemness and tumor growth (45–47). In one case, the GPNMB growth effect depended on IL-33/ST2 signaling in MCA-1 fibrosarcoma cell cultures (53). However, the basal ESC program for hyperplasia/metaplasia is unaffected by ST2 gene knockout in the SeV mouse model (27). Instead, basal cell growth depends on nuclear function of IL-33 as a cell cycle checkpoint. This action is complemented by secreted IL-33 that is required for downstream production of IL-13 by macrophages and type 2 innate lymphoid cells and consequent inflammation and mucus production (41). The present findings establish GPNMB function in a non-cancer stem cell context that promotes inflammatory disease. The data also indicate that GPNMB is context dependent with the present function set in the matrix of timing for GPNMB^+^ moDC and IL-33^+^ basal ESC interaction at 1–2 weeks after viral infection. As developed below, this signaling mechanism is demonstrable across mouse and human cell models, suggesting translation to clinical conditions.

Relatedly, GPNMB expression later in the disease process, primarily in M2-alveolar macrophages, could also contribute to basal ESC reprogramming toward lung disease. In the present model, early moDC and later alveolar macrophage production of GPNMB coincides with progressive basal ESC hyperplasia/metaplasia and then immune activation that might feed forward to promote immune cell infiltration and inflammation. This relatively prolonged time course also implies a likelihood of detecting a GPNMB^+^ alveolar macrophage signature in chronic lung disease even long after the time of a previous infection. Indeed, this might be the case given the prominence of CD68^+^ macrophages found in clinical samples of lung tissue in long-term COVID-19 (28). These findings thereby provide the potential for useful biomarker guidance for precise therapy in PVLD and related conditions.

Third, the present analysis suggests that selective GPNMB signaling via the CD44 receptor controls basal ESC growth in the context of PVLD. As introduced in Results, these findings fit with reports of GPNMB/CD44 signaling in mesenchymal, neural, and cancer cells (51–53). The downstream signaling pathway still needs to be fully defined, but our work on CCL5/ERK/AKT, EGFR, and MAPK13 in human cell and animal models provides a well-characterized set of kinase signaling candidates relevant to PVLD (42, 58–60). Linking GPNMB to these downstream effectors is key to the development of a new therapeutic strategy to precisely modify stem cell function and consequent PVLD. In the present experiments, Abs to block GPNMB or CD44 were effective for at least 35 days despite an expected half-life of 6–8 days in mice (61), reflecting the advantage of targeting a renewable ESC population as done for cancer therapies. This signaling pathway is also implicated in fibrosis as found previously by us in respiratory viral infections (39, 40) and by others working on the lung and other sites of disease (62–65). These findings create an opportunity for intervention in this post-viral phenotype as well with an alternative focus on growth and differentiation of mesenchymal stem cells. In each case, GPNMB signaling might depend on cell-cell contact and cell-remote actions, given that GPNMB is active in cell-surface and cleaved-secreted forms (66).

Fourth, in relation to stem cell function, the present work provides a mechanism for basal ESC growth and immune activation in vivo as a basis for studies of 3D organoid culture in vitro. In particular, a specialized immune niche supplies GPNMB directly to the basal ESC program for growth and motility (evidenced by organoid formation), immune activation (based on chemokine/cytokine production for direction of immune cell infiltration and type 2 cytokine production), and mucinous differentiation (driving basal to mucous cell transition). This matrix delivers the key phenotypes responsible for the mortality and morbidity of chronic lung diseases and a substrate to define targets for disease modification. This goal is initiated here for anti-GPNMB Ab blockade and in parallel work on small-molecule inhibition of a stress kinase (MAPK13) that is also linked to basal ESC function (42, 60, 67). In that regard, both *Wfdc21* and GPNMB function appears to be selective for basal ESC versus AT2 cell reprogramming, in contrast to earlier work on TLR3^+^ moDCs that increased AT2 cell growth as well (49). This cell selectivity might better avoid off-target effects that could compromise alveolar repair and function in the setting of lung injury. Nonetheless, TLR3 or related pathogen recognition receptors might provide a link between viral infection/replication and sentinel cell activation.

In sum, the present results provide evidence of *Wfdc21*-dependent moDCs as a distinct immune cell niche for basal ESC growth and immune activation in general and the route to type 2 inflammation and mucus production in particular in PVLD. The moDC niche function is precisely timed and programmed for GPNMB signaling to CD44 on basal ESCs. The results fill a key gap in understanding how viral infection and sentinel immune cells might lead to stem cell reprogramming as a general response to host epithelial cell injury due to infection and other damaging and immune-activating agents. The observations also provide instructions for a specific therapeutic strategy to precisely and timely interrupt this signaling pathway using agents that attenuate moDC function at the level of *Wfdc21* and/or GPNMB and downstream basal ESC function at the level of GPNMB/CD44 signaling interactions. This approach is exemplified here using anti-GPNMB Ab that is already proven safe when it is applied to stem cell dysfunction in cancer (68). These strategies to correct reprogramming of a renewable stem cell population offer the potential for long-lasting disease modification as found in the present study.

## Methods

### Sex as a biological variable.

Results from male and female mice were pooled since no significant differences were found between sexes as reported initially (69) and confirmed recently (40) and in the present experiments.

### Study design.

This study used a multidisciplinary approach to identify the upstream cell and molecular control of basal ESCs in an experimental model of lung disease. First, a subtractive RNA sequencing approach was used to identify a molecular candidate for the proposal that moDCs participated in pathogenesis of PVLD. Genetic knockout of this candidate was used to define functional biology in a corresponding mouse model. Corresponding analysis of scRNA-Seq was engaged to define moDC participation and possible moDC products to drive basal ESC growth and activation. Function was assigned in vivo using Ab blockade. Further analysis of scRNA-Seq data sets for signal transduction candidates was also validated in vivo with Ab blockade. This scheme was extended to organoid cultures to assign function directly to basal ESCs. Finally, the results were extended to human cell samples to define clinical relevance to basal ESC reprogramming. In each case, power analysis and past experience with the experimental models were used to provide guidelines for adequate numbers of animals or cell-based assays to assure probability of statistical significance at *P* less than 0.05 and replicates for adequate reproducibility.

### Mouse models.

Male and female wild-type (WT) C57BL/6J mice (000664; 5–6 weeks of age) were obtained from The Jackson Laboratory. The *Wfdc2* gene–knockout mice (*Wfdc21^−/−^*) were generated in the C57BL/6J background using CRISPR/Cas9 technology with guide RNAs (gRNAs) that were designed to target upstream of exon 1 and downstream of the last exon 3 (including the 3-UTR) based on sequence to minimize any off-target effect. Synthetic gRNAs were purchased from IDT Technologies and complexed with recombinant Cas9 protein before nucleofection in Neuro-2a cells for validation. The gRNAs that cleaved the whole *Wfdc21* gene were chosen to generate the knockout allele. The ribonucleoprotein complex containing Cas9 protein (1 μg/μL) and gRNA (0.3 μg/μL) was then electroporated into single-cell embryos of C57BL/6J mice, and 25–30 eggs were transferred into each pseudopregnant female to generate founder mice. Knockout founders were identified by analysis of the PCR-amplified target region (forward primer 5′-GCTTACTGTCTCCTCAACAGG and reverse primer 5′-GTGCACTATTTTAGGTGGTTTAAGGCTATG) using next-generation sequencing to identify out-of-frame indels. Heterozygous F_1_ mice were obtained from matings between founders and WT mice, and homozygous F_2_ mice were obtained through sibling matings of F_1_ mice. The resulting *Wfdc21^−/−^* mice exhibited complete loss of *Wfdc21* RNA expression and reproduced and developed normally relative to WT control mice.

All mice were maintained and cohoused in a barrier facility using cages fitted with micro-isolator lids. Sendai virus (SeV; Sendai/52 Fushimi strain, ATCC VR-105) was obtained from ATCC and prepared and titered by plaque-forming assay and quantitative PCR (qPCR) assay as described previously (36). Influenza A virus (IAV; strain A/PR/8/34) was obtained from Jacco Boon (Washington University). Mice were infected with SeV (2.6 × 10^5^ PFU) or IAV (50 PFU) as described previously (39, 40). Virus or an equivalent amount of UV-inactivated virus or PBS alone was delivered intranasally in 30 μL of PBS under ketamine/xylazine anesthesia at 5–6 weeks of age. Viral titers for stock solutions and lung infections were monitored by qPCR assay with primers for SeV-*NP* RNA as defined previously and in Supplemental Table 2 using SeV-*NP*–expressing plasmids as an internal standard (40). Ab blockade was performed with goat anti-GPNMB Ab (R&D Systems, AF2330), rat anti-CD44 mAb (clone IM7; Bio X Cell, BE0039), or corresponding control IgG (R&D Systems, AB-108-C, and Bio X Cell, BE0130 and BE0094, respectively) administered by intraperitoneal injection.

### Bulk RNA-Seq.

Lung tissue RNA was purified as described previously (70) from 3 mice per sample condition (12 or 21 days after SeV infection or PBS control challenge) and subjected to RNA-Seq using a NovaSeq 6000 with S4 flow cell (Illumina) in the Genome Technology Access Center in the McDonnell Genome Institute (Washington University). For RNA-Seq analysis, gene and transcript levels were quantified using the RSEM (version 1.3.2; https://bmcbioinformatics.biomedcentral.com/articles/10.1186/1471-2105-12-323) pipeline, and the estimated counts were stored as the appropriate raw data to feed into the edgeR (version 3.24.3; https://bioconductor.org/packages/release/bioc/html/edgeR.html), limma (version 3.38.3; https://www.ncbi.nlm.nih.gov/pubmed/25605792; https://www.bioconductor.org/packages/release/bioc/vignettes/limma/inst/doc/usersguide.pdf), and voom (version 3.38.3; https://www.ncbi.nlm.nih.gov/pubmed/24485249) pipeline. The list of Ensembl gene IDs was annotated by BioMart (version 2.38.0), which queried BioMart databases (https://bioconductor.org/packages/release/bioc/html/biomaRt.html) to retrieve additional information including Entrez Gene identifiers for gene symbol and chromosome. A DGEList appropriate for our data was declared, and filterByExpr function in edgeR was used to maintain 35% of the genes in the gene list by filtering out lowly expressed genes automatically and in a principled way. The data were then normalized to account for variability in sequencing depth, library size (71), and other external factors that are not of biological interest. After trimmed mean of M values (TMM) normalization (72), unsupervised hierarchical clustering with the hclust function in base R (version 3.6.3) and principal component analysis (PCA) with the prcomp function were conducted to identify treatment and control clusters. The correlations between replicate samples were also examined, with the lowest value of 0.94. We invoked voom software to compute precision weights and fitted linear models to the data using the lmFit function. Then, we refitted the model using the contrasts.fit function and computed moderated t and F statistics using the eBayes and treat functions, respectively. We chose the outputs from the treat function for a more stringent definition of statistical significance to test against a fold change threshold. Heatmaps were generated with heatmap.2 function in gplots (version 3.1.0; https://biocorecrg.github.io/CRG_RIntroduction/heatmap-2-function-from-gplots-package), based on subsets of the top 100 most significant genes sorted separately by the F statistic and fold change. We also generated scatterplots with ggplot2 (version 3.3.2) with the top genes ranked by fold change and annotated when relevant to the present model.

### Real-time PCR assay.

RNA was purified from homogenized lung tissue using Trizol (Invitrogen) or from isolated cells with the RNeasy Mini Kit (Qiagen) and was used to generate cDNA with the High-Capacity cDNA Archive kit (Life Technologies). We quantified target mRNA and viral RNA levels using real-time qPCR assay with specific fluorogenic probe-primer combinations and Fast Universal PCR Master Mix systems (Applied Biosystems) with mouse-specific forward and reverse primers and probes as described previously (41) and in Supplemental Table 2. All samples were assayed using the QuantStudio 6 Fast Real-Time PCR System and analyzed using Fast System Software (Applied Biosystems). All real-time PCR data were normalized to the level of *Gapdh* mRNA. Values were expressed as fold change based on the ΔΔCt method as described previously (73).

### Flow cytometry and FACS.

As in our previous reports for flow cytometry and FACS (27, 41, 49), single-cell suspensions were generated from minced lung tissue that was subjected to collagenase (Liberase TM Research Grade, Roche), hyaluronidase (MilliporeSigma), DNase I (MilliporeSigma), and Dispase II (Roche) digestion for 45 minutes at 37°C and then treated with ACK buffer (Lonza) to remove red blood cells. Following FcR blockade, lung cell suspensions were incubated with labeled Abs and were sorted using a Sony SY3200 Synergy high-speed cell sorter. The following Abs were used: anti–mouse CD31 (clone MEC 13.3; BD Biosciences), anti–mouse CD45 (clone 30-F11; BD Biosciences), anti–mouse EpCAM (clone G8.8; BioLegend), anti-AQP3 (Abcam, Ab125219), anti-mCD11b (clone M1/70; eBioscience), anti–Ly-6C (clone al-21; BD Biosciences), anti–Ly-6G (clone 1a8; BD Biosciences), anti–mouse F4/80 (clone BM8; eBioscience), anti–mouse CD11c (clone HL3; BD Biosciences), and anti–mouse Siglec-F (clone E50-2440; BD Biosciences). Anti-AQP3 Ab was labeled using the Zenon antibody labeling kit (Molecular Probes). FACS results were plotted and analyzed using FlowJo software (Tree Star). For the present experiments, the following cell populations were isolated: moDC (Ly-6G^–^, CD11c^+^, Siglec-F^–^, CD64^+^, and CD11b^+^), tissue monocyte (Ly-6G^–^, CD11c^–^, Siglec-F^–^, SSC^lo^, CD64^+^, and CD11b^+^), alveolar macrophage (Ly-6G^–^, CD11c^+^, Siglec-F^+^, CD64^+^, and CD11b^–^), CD11b^+^ DC (Ly-6G^–^, CD11c^+^, Siglec-F^–^, CD64^–^, and CD11b^+^), CD103^+^ DC (Ly-6G^–^, CD11c^+^, CD64^–^, Siglec-F^–^, CD11b^–^, and CD103^+^), and epithelial cell (CD45^–^, CD31^–^, and EpCAM^+^), using flow cytometry schemes (Supplemental Figure 1) based on those described previously (27, 41, 49).

### Histology and immunodetection.

Lung tissue was fixed with 10% formalin, embedded in paraffin, cut into 5-μm sections, and adhered to charged slides. Sections were stained with PAS and hematoxylin as described previously (27, 40). For immunostaining, sections were deparaffinized in Fisherbrand CitriSolv (Fisher), hydrated, and heat-treated with antigen unmasking solution (Vector Laboratories Inc.). Immunostaining was performed with the commercially available primary Abs detailed in Supplemental Table 1. Primary Abs were detected with secondary Abs labeled with Alexa Fluor 488 or Alexa Fluor 594 (Thermo Fisher Scientific) followed by DAPI counterstaining. Slides were imaged by light microscopy using a Leica DM5000 B and by immunofluorescent microscopy using an Olympus BX51, and staining was quantified in whole-lung sections using a NanoZoomer S60 slide scanner (Hamamatsu) and ImageJ software (NIH) as described previously (27, 40). To correlate immunostaining with protein production, levels of GPNMB were also determined in bronchoalveolar lavage (BAL) fluid and lung tissue homogenates obtained as described previously (27) and quantified using the DuoSet ELISA kit (R&D Systems, DY2330).

### Single-cell RNA sequencing.

Single-cell RNA sequencing (scRNA-Seq) was performed as described previously (27) but in this case using whole-lung cell digests instead of lung cells subjected to FACS purification. Thus, total lung cells were isolated with the same enzymatic dissociation procedure at 12, 21, and 49 days after SeV infection or control PBS challenge. In both cases, cellular mRNA was purified and subjected to scRNA-Seq using the 10x Genomics platform. Next-generation sequencing data were processed with the Cell Ranger set of analysis pipelines (version 2.1.0; https://www.10xgenomics.com/support/software/cell-ranger/latest) as follows: First, FASTQ files were generated for each sample by demultiplexing of the sequencer’s base call files. Then, single-cell gene count matrices were generated by running of “cellranger count” with mm10 transcript reference per sample, and all sample data were aggregated with “cellranger aggr.” In this process, sequence depth was normalized by subsampling reads from higher-depth data. The aggregated gene expression matrix contained 80,147 cells in total. Next, the gene expression matrix was processed with the R package Scater 1.6.3 (74). Before Scater normalization, we removed outlier cells with an extremely large or small number of unique molecular identifiers (UMIs) (UMI > 20,000 or UMI < 500) or a large mitochondria/UMI ratio (>0.2). With these quality checks, 88.8% of the cells passed to enter further analysis. We also removed genes with low average expression (mean UMI count < 0.01). Cell cycling scores were calculated for each cell using the cyclone algorithm in the R package Scran 1.6.9 (75). The gene expression matrix was then normalized with Scater, which includes size normalization and log transformation. Normalized gene expression matrix data were imported to the R package Seurat 2.3.4 (76) without further normalization and were directly subjected to scaling and undesired variation removal wherein process data variations correlated with UMI count and percentage of mitochondria were regressed out. Additional data analysis for clustering, dimensional reduction, marker identification, and visualization was performed with Seurat. For dimensional reduction, PCA and t-distributed stochastic neighbor embedding (t-SNE) were performed. Genes with high variable expression were selected and used for PCA. The first 40 PCs were selected as important PCs based on the distribution of standard deviation for each PC. The 40 PCs were used for clustering and t-SNE. A shared nearest neighbor modularity optimization-based clustering algorithm was used for clustering. The clusters were manually annotated by marker gene expression. Marker genes for each cluster were identified with the FindAllMarkers function in Seurat.

Cell-cell interactions were identified using the CellChat package (version 1.1.3; https://github.com/sqjin/CellChat), which integrates gene expression with ligand and receptor signaling databases. For this analysis, the present scRNA-Seq data set was processed using the commands for “create CellChat” “identifyOverExpressedGenes,” “identifyOverExpressedInteractions,” and “computeCommunProb.” The analysis was run under the R package to detect significant interactions between basal cells and tissue monocytes versus moDCs.

### Mouse and human cell culture.

For 3D organoid cultures, mouse lung basal epithelial cells from FACS (CD31^–^CD45^–^EpCAM^+^AQP3^+^ as described above) were resuspended in Small Airway Epithelial Cell Growth Medium (SAGM, Lonza) mixed 1:1 with growth factor–reduced Matrigel (BD Biosciences) and plated at 3 × 10^3^ cells per 100 μL in 24-well Transwells (Corning). For the first 5 days, SAGM was added with 10 μM ROCK inhibitor (MilliporeSigma) but without FBS. After 5 days, SAGM was added with 10% FBS but without ROCK inhibitor. The medium was changed every 2–3 days. Organoids were cultured for 14 days for counts to monitor formation and were harvested from Matrigel using cell recovery solution (BD Biosciences). Harvested organoids were used to isolate mRNA for real-time qPCR assay (Supplemental Table 2). In some experiments, cultures were incubated with recombinant GPNMB (R&D Systems, 2330) with or without anti-CD44 Ab (Bio X Cell) for 14 days at 37°C. For corresponding studies of human 3D organoids, human tracheobronchial epithelial cell (hTEC) cultures were established as described previously (43) and then maintained for 14 days at 37°C under the same conditions as for mouse cells except that EGF was not added to the culture medium to slow and better detect organoid formation after incubation with GPNMB (R&D Systems, 2550). Harvested human organoids were also subjected to real-time qPCR assay (Supplemental Table 3).

### Statistics.

All data presented in bar graph formats were expressed as mean ± SEM. For these data, statistical differences between means for sample conditions were assessed using 1-way ANOVA with Tukey’s correction for multiple comparisons. For all data, the significance threshold was set at *P* less than 0.05. The number of mice for each condition and the number of replicate experiments are defined in the figure legends.

### Study approval.

Animal husbandry and experimental procedures were approved by the Institutional Animal Care and Use Committees of Washington University School of Medicine in accordance with the guidelines of the NIH. Use of human tissue was reviewed and approved by the Institutional Review Board of Washington University School of Medicine, and written informed consent was obtained from study participants.

### Data and materials availability.

Mouse models reported here are available through material transfer agreement from Washington University. The RNA-Seq and scRNA-Seq data were deposited in the NCBI’s Gene Expression Omnibus (GEO) under series accession numbers GSE271147 (RNA-Seq) and GSE178517 (scRNA-Seq).

## Author contributions

KW performed mouse and cell experiments and analyzed scRNA-Seq data. YZ generated *Wfdc21^–/–^* mice. HYD performed immunostaining experiments. DM performed mouse and cell experiments. KS performed mouse experiments. SRA registered human lung samples. KY analyzed RNA-Seq data. ECC identified and analyzed autopsy samples. SLB obtained and provided human airway epithelial cells. DEB obtained and provided human clinical samples. CMH and MEH analyzed bulk RNA-Seq data. MJH directed the project and wrote the manuscript.

## Supplementary Material

Supplemental data

Supporting data values

## Version 1 07/25/2024

In-Press Preview

## Version 2 09/17/2024

Electronic publication
