# Supplementary material for: A correctable immune niche for epithelial stem cell reprogramming and post-viral lung diseases
Source: J Clin Invest. 2024 Jul 25;134(18):e183092. doi: 10.1172/JCI183092 (PMC11405052; doi:10.1172/JCI183092)
Supplement: Supplemental data [file jci-134-183092-s158.pdf]

Supplemental Materials

A correctable immune niche for epithelial stem-cell reprogramming and post-viral lung diseases

Kangyun Wu[1], Yong Zhang[1], Huiqing Yin-DeClue[1], Kelly Sun[1], Dailing Mao[1], Kuangying Yang[1], Stephen R. Austin[5], Erika C. Crouch[2], Steven L. Brody[1], Derek E. Byers[1], Christy M. Hoffmann[1], Michael E. Hughes[1,3*], and Michael J. Holtzman[1,4]

[1]Pulmonary and Critical Care Medicine, Department of Medicine, [2]Department of Pathology and Immunology, [3]Department of Genetics, [4]Department of Cell Biology and Physiology, Washington University School of Medicine, Saint Louis, MO 63110

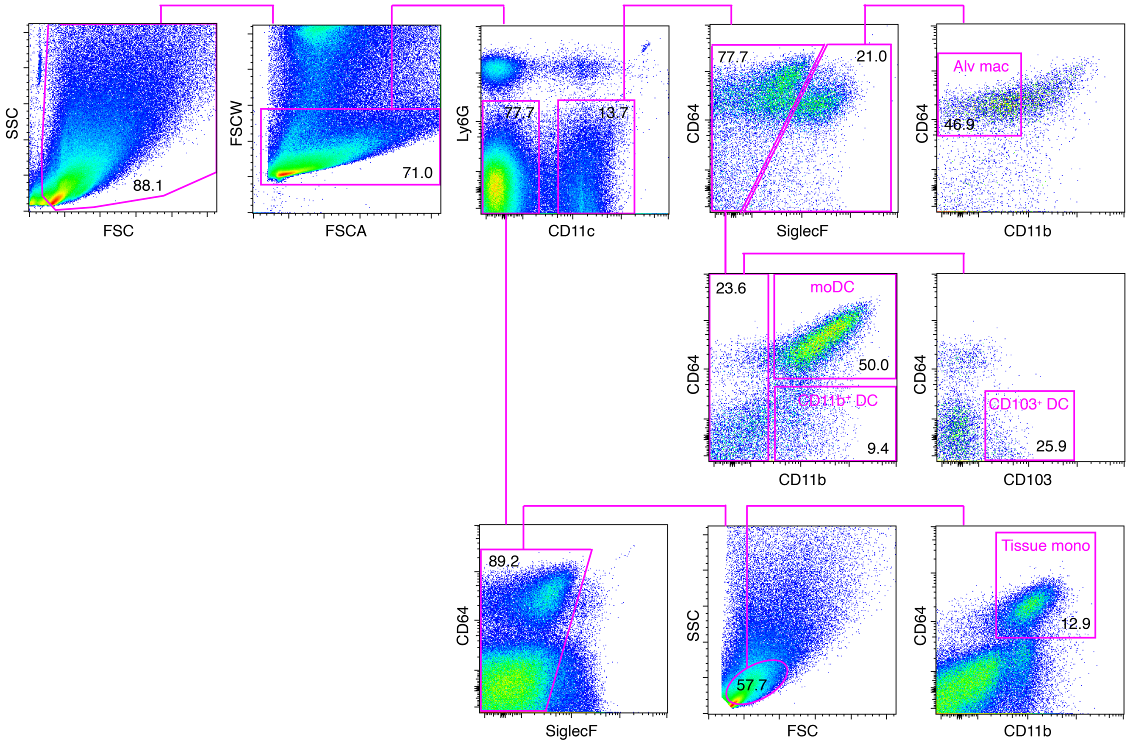

Supplemental Figure 1. Flow cytogram scheme for immune cell populations. Lung cells were isolated and separated into populations as described in Methods. Abbreviations: SSC, side scatter; FSC, forward scatter; FSCW, forward scatter width; FSCA, forward scatter area.

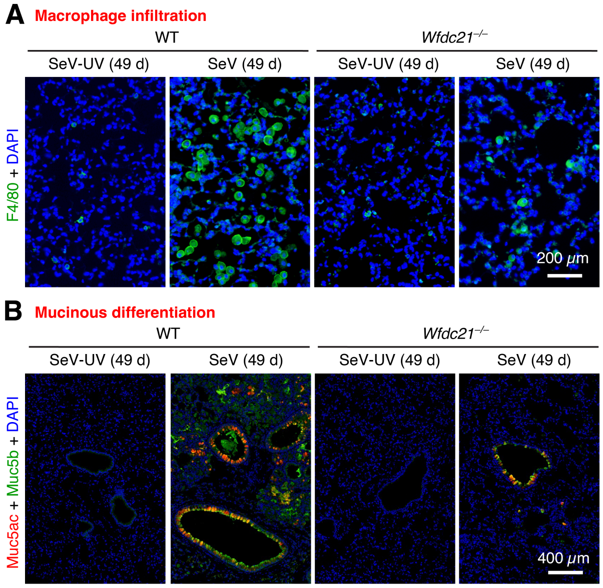

Supplemental Figure 2. Effect of the Wfdc21-dependent moDC niche on PVLD. A, Immunostaining for F4/80 with DAPI counterstaining in lung sections from WT and *Wfdc21*$^{-/-}$ mice at 49 d after SeV infection or SeV-UV control. **B**, Immunostaining for Muc5ac and Muc5b with DAPI counterstaining in lung sections for conditions in (A).

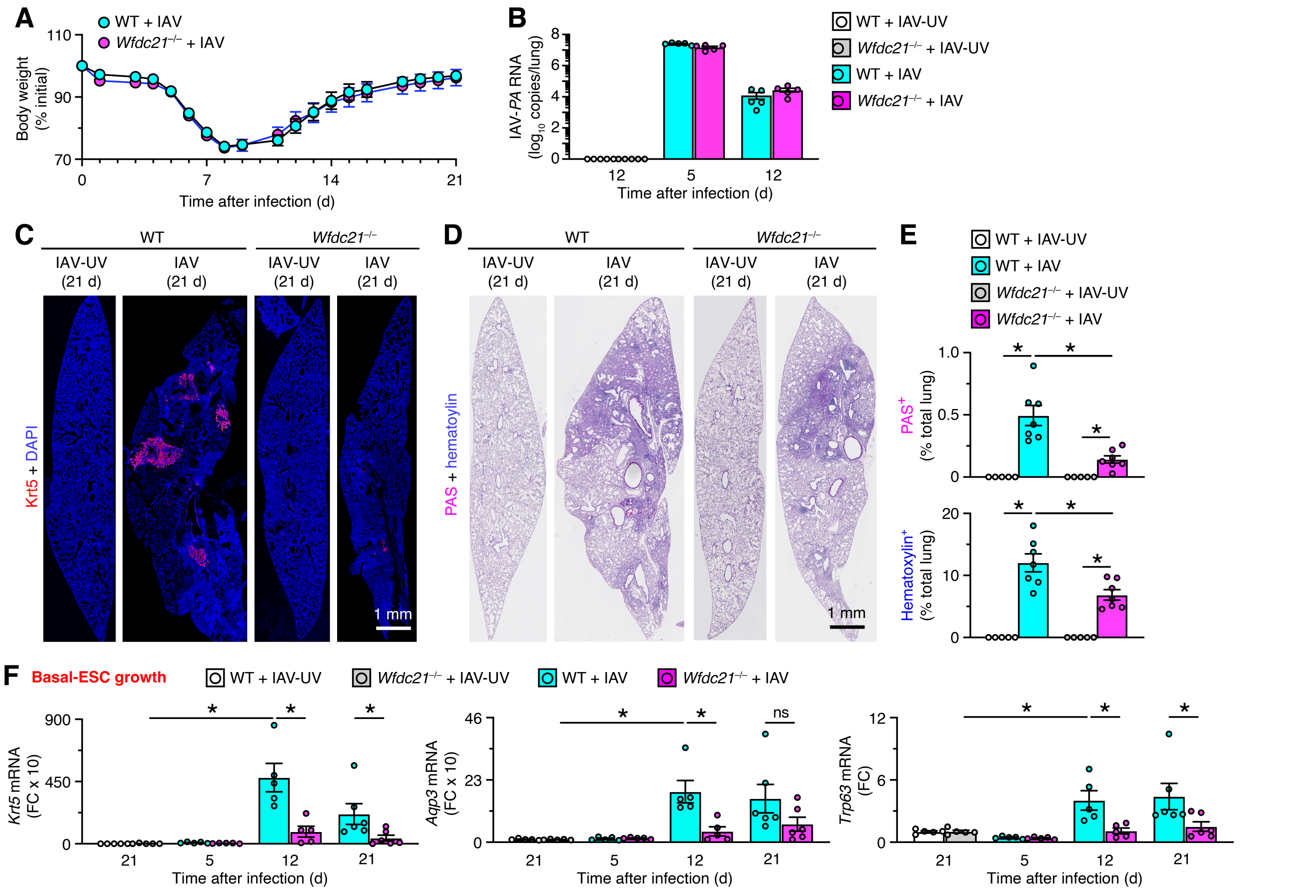

Supplemental Figure 3. Effect of the Wfdc21-dependent moDC niche on PVLD after influenza A virus (IAV) infection. A, Body weights for WT and *Wfdc21*–/– mice at 0-21 d after influenza A virus (IAV, PR8 strain) infection. **B,** IAV-*PA* RNA levels in lung tissue from WT and *Wfdc21*–/– mice at 0-12 d after IAV infection. **C,** Immunostaining for Krt5 with DAPI counterstaining in lung sections from WT and *Wfdc21*–/– mice at 21 d after IAV infection or IAV-UV control. **D,** PAS and hematoxylin staining of lung sections for conditions in (C). **E**, Quantitation of staining for (D). **F,** Lung tissue levels of mRNA biomarkers at 0-21 d after IAV infection and 21 d after IAV-UV control to track basal-ESC growth. Values represent mean ± s.e.m. (n=5-10 mice per condition). *P <0.05 by ANOVA and Tukey correction.

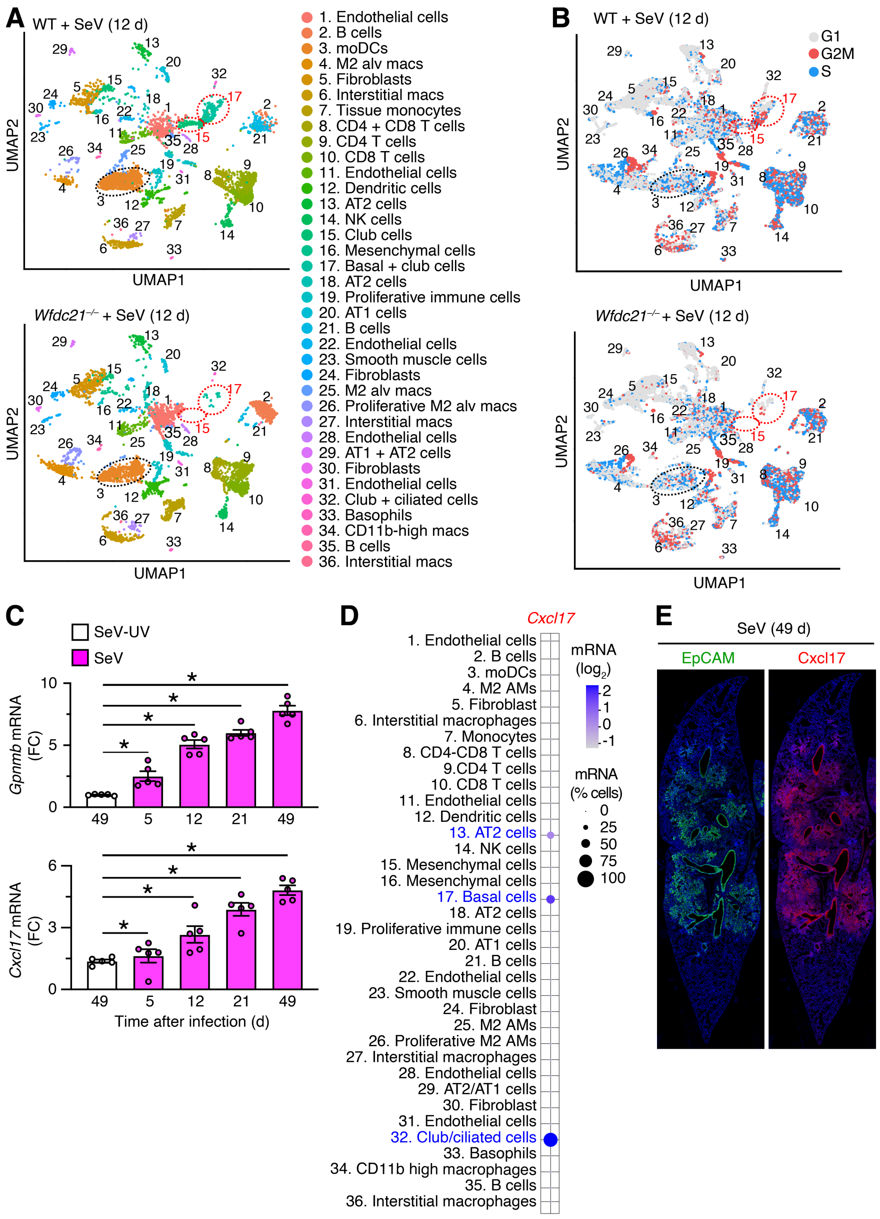

Supplemental Figure 4. Identification of *Gpnmb* gene expression in moDCs in concert with basal-ESC proliferation. A, Gene cluster assignments for WT and *Wfdc21*$^{-/-}$ at 12 d after SeV infection. **B,** Cell cycle analysis of gene expression for conditions in (A). Basal and basal-lineage club cells (clusters 15 and 17) are highlighted with red-dashed circles and moDCs (cluster 3) with black-dashed circles. **C,** Levels of *Gpnmb* and *Cxcl17* mRNA in lung tissue at 5-49 d after SeV infection and 49 d after SeV-UV control. **D,** Dot plot analysis of scRNAseq of total lung cells combined from WT mice at 12 and 21 d after SeV infection and PBS control. **E**, Immunostaining for EpCAM and Cxcl17 in lung sections from mice at 49 d after SeV infection. Values represent mean ± s.e.m. (n=5 mice per condition). **P* <0.05 by ANOVA and Tukey correction.

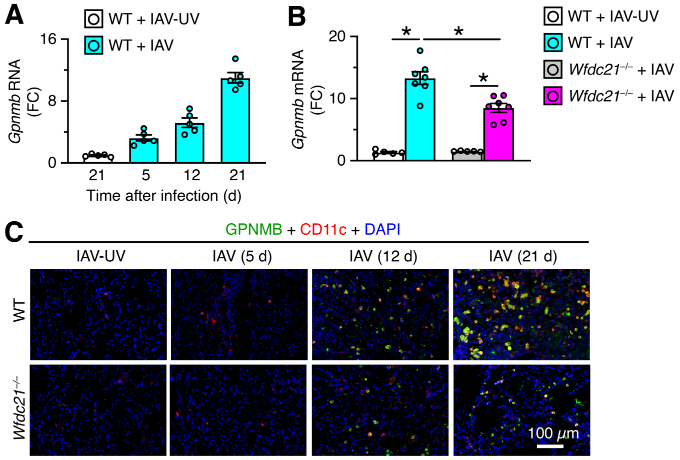

Supplemental Figure 5. Wfdc21-dependent induction of *Gpnmb* gene expression in moDCs and macrophages is also found after IAV infection. A, Levels of *Gpnmb* mRNA in lung tissue at 5-21 d after IAV infection and 21 d after IAV-UV control. **B,** *Gpnmb* mRNA levels in lung tissue from WT and *Wfdc21*$^{-/-}$ mice at 21 d after IAV infection or IAV-UV control. **C,** Immunostaining for GPNMB and CD11c with DAPI counterstaining in lung sections from WT and *Wfdc21*$^{-/-}$ mice at 5-21 d after IAV infection and 21 d after IAV-UV control. Values represent mean ± s.e.m. (n=3-6 mice per condition). **P* <0.05 by ANOVA and Tukey correction.

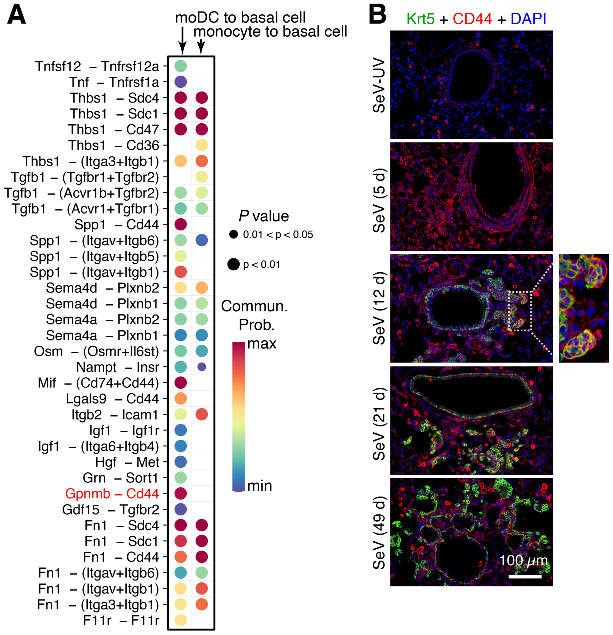

Supplemental Figure 6. Identification of a Gpnmb-CD44 signaling pathway in PVLD. **A**, Analysis of interaction pathways for lung moDCs versus tissue monocytes to basal epithelial cells using the CellChat package. **B**, Immunostaining for Krt5 and CD44 with DAPI counterstaining in lung sections from WT mice at 5-49 d after SeV infection or SeV-UV control. Inset, 4x magnification.

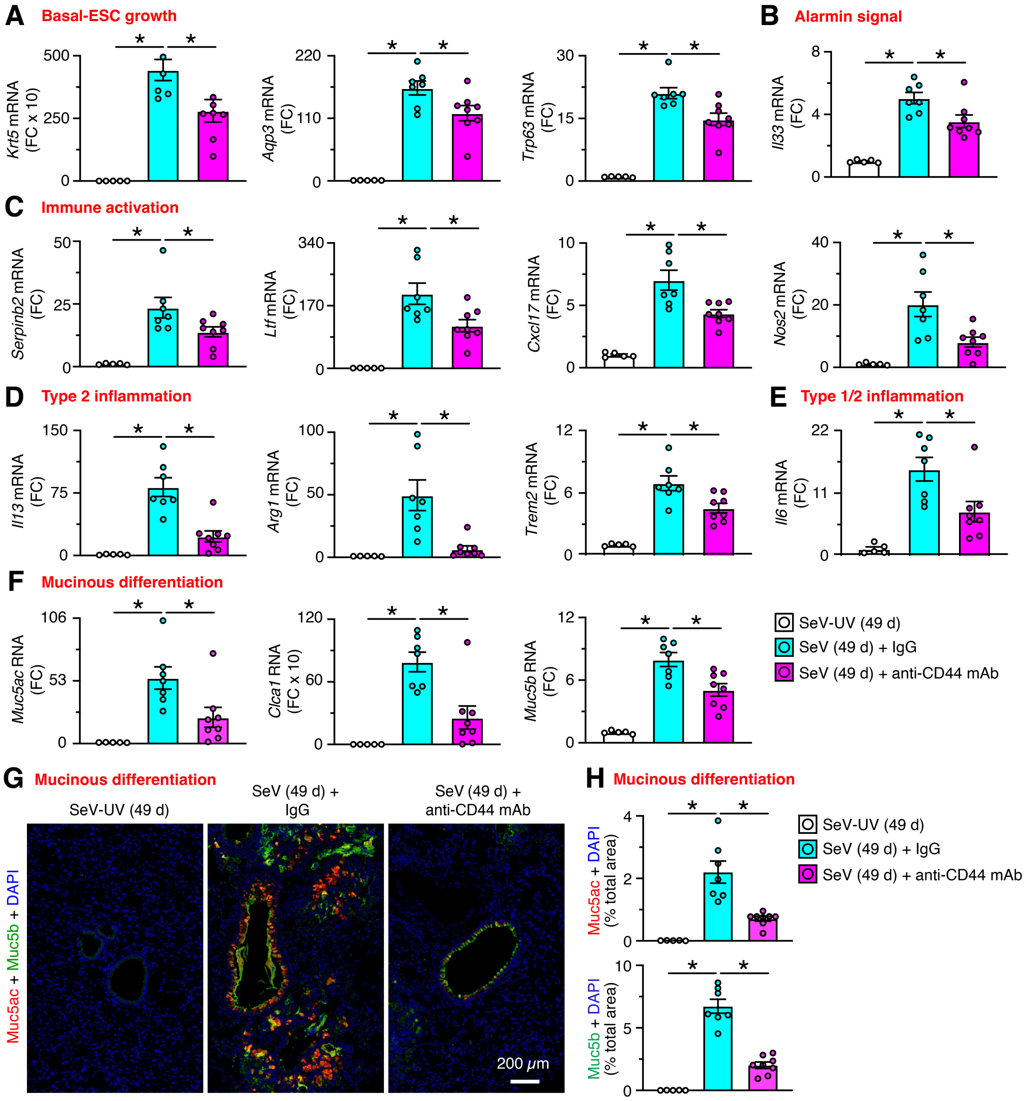

Supplemental Figure 7. Effect of anti-CD44 Ab blockade on biomarkers for PVLD. For the protocol scheme for anti-CD44 Ab or control IgG treatment of mice shown in Fig. 7A: **A-F,** Lung tissue levels of mRNA biomarkers in mice at 49 d after SeV or Sev-UV to track basal-ESC growth (A), alarm signal (B), immune activation (C), type 2 inflammation (D), type 1/2 inflammation (E), and mucinous differentiation (F). **G**, Immunostaining for Muc5ac and Muc5b with DAPI counterstaining in lung sections for conditions in (A-E). **H**, Quantitation of staining in (F). Values represent mean ± s.e.m. (n=6-10 mice per condition). **P* <0.05 by ANOVA and Tukey correction.

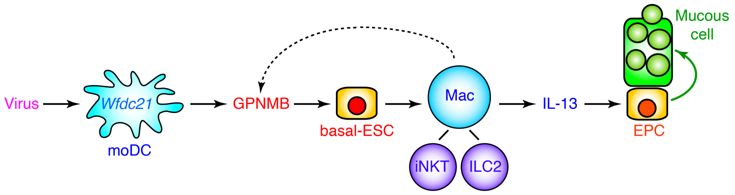

Supplemental Figure 8. Scheme for immune-cell niche driven events in PVLD. In this scheme, typified by SeV infection, sequential steps include moDC infiltration and differentiation that requires *Wfdc21* expression; moDC production of Gpnmb that stimulates basal-ESC growth and immune activation (marked by chemokine Cxcl17 and alarmin IL33). Subsequent infiltration and interaction of macrophage (Mac) with invariant iNKT (iNKT) cell and ILC2 populations results in cytokine IL-13 production and in turn basal-epithelial progenitor cell (EPC) differentiation to mucous cells. In addition, macrophage production of GPNMB provides for feedback amplification of this pathway. Each of these events is identified in the present mouse model of PVLD and is also detected in hTEC culture models and clinical samples of lung tissue from comparable remodeling disease in humans.

Supplemental Table 1. Antibodies for mouse tissue immunostaining.

Target Protein	Antibody Type	Vendor	Catalogue #
CD11c	Rabbit mAb	Cell Signaling	97585/45581
CD44	Rabbit mAb	Abcam	Ab189524
CD68	Rabbit mAb	Cell Signaling	76437
Cxcl17	Sheep pAb	R&D systems	AF4270
EpCAM	Rabbit mAb	Cell Signaling	93790
F4/80	Rabbit mAb	Cell Signaling	700076
GPNMB	Mouse mAb	Proteintech	66926
IL-33	Goat mAb	R&D systems	AF3626
Ki-67	Rabbit mAb	Cell Signaling	12202
Krt5	Chicken mAb	Biolegend	905904
Muc5ac	Mouse mAb (45M1), biotinylated	ThermoFisher Scientific	MA5-12175
Muc5b	Rabbit pAb	Abcam	Ab87376
Sftpc	Rabbit pAb	Abcam	Ab90716

Supplemental Table 2. Primer and probe sequences and sources for real-time quantitative PCR assays for mouse mRNA and viral RNA levels.

Target gene		ID/Sequence
Aqp3	F	5'-AAGTTGATGGTGAGGAAGCC-3'
	R	5'-ATGCTTCACATCCGCTACC-3'
	P	5'-TCCTTGTGATGTTTGGCTGTGGC-3'
Arg1	F	5'-AGTGTTGATGTCAGTGTGAGC-3'
	R	5'-GAATGGAAGAGTCAGTGTGGT-3'
	P	5'-ACAGTCTGGCAGTTGGAAGCATCT-3'
Clca1	F	5'-AGACCATTGTTCTGAACCTGATCCGAAG-3'
	R	5'-ACCGGCTGCCGCTAAAGAGCTTGAG-3'
	P	5'-AAATGACAGGAGGCCTGCAGACATA-3'
Cxcl17	F	5'-CCTTCTGTTGCTTCCAGTGA-3'
	R	5'-TTCCAAGAGCCACCTCCTA-3'
	P	5'-TCATGTCCATGGTCTTCAGCAGCC-3'
Gapdh	F	5'-GTGGAGTCATACTGGAACATGTAG-3'
	R	5'-AATGGTGAAGGTCGGTGTG-3'
	P	5'-TGCAAATGGCAGCCCTGGTG-3'
Il13	F	5'-GGTGCCAAGATCTGTGTCTC-3'
	R	5'-CCACACTCCATACCATGCTG-3'
	P	5'-AAGACCAGACTCCCCTGTGCAAC-3'
Il33	F	5'-TCATGTTCACCATCAGCTTCT-3'
	R	5'-GTGCTACTACGCTACTATGAGTC-3'
	P	5'-ACCGTCGCCTGATTGACTTGCA-3'
Il6	F	5'-AGCCAGAGTCCTTCAGAGA-3'
	R	5'-TCCTTAGCCACTCCTTCTGT-3'
	P	5'-ATTTCCAATGCTCTCCT-3'
Krt5	F	5'-AGATCGCCACCTACAGGAA-3'
	R	5'-TCCGTAGCCAGAAGAGACA-3'
	P	5'-CCCACTCAGCCTGCACTCCTC-3'
Ltf	F	5'-CCCAAGACCACAGACATGAG-3'
	R	5'-CAGAATTTGACACAGCACACC-3'
	P	5'-TTAGCTAGACAGAGTCCAAGGGCCT-3'
Muc5ac	F	5'-TACCACTCCCTGCTTCTGCAGCGTGTCA-3'
	R	5'-ATAGTAACAGTGGCCATCAAGGTCTGTCT-3'
	P	5'-TATACCCCTTGGGATCCATCATCTACA-3'
Muc5b	F	5'-GCATCATCAACAGTGCAACG-3'
	R	5'-GCAGAAACACTCGCAGTCA-3'
	P	5'-ATGCACACAGGCTTC-3'
Nos2	F	5'-GACTGAGCTGTTAGAGACACTT-3'
	R	5'-CACTTCTGCTCCAAATCCAAC-3'
	P	5'-CCCAAGCGTGAGGAG-3'
Serpinb2	F	5'-GAGCATCTCATCAACATTGGC-3'
	R	5'-GTTCTCTGGGTTTCTTGTGGT-3'
	P	5'-CATCTGCTGTTCAGTGTTACCCCCA-3'
Trem2	NA	Mm00451744_m1
Trp63	F	5'-GAGATGAGGAGGTGAGGAGAA-3'
	R	5'-ACAGACTGCAGCATTGTCAG-3'
	P	5'AGATGGCATGTCGGAACTGTTCAGG-3'
SeV	F	5'-GGCGGTGGTGCAATTGAG-3'
	R	5'-CATGAGCTTCTGTTTCTAGGTCGAT-3'
	P	5'-AGCTCTAGACAATGCC-3'
M (A/PR/8/34)	F	5'-AGATGAGTCTTCTAACCGAGGTCG-3'
	R	5'-TGCAAAAACATCTTCAAGTCTCTG -3'
	P	5'-TCAGGCCCCCTCAAAGCCGA -3'

Supplemental Table 3. Primer and probe sequences and sources for real-time quantitative PCR assays for human mRNA levels.

GAPDH	F	5'-TGTAGTTGAGGTCAATGAAGGG-3'
	R	5'-ACATCGCTCAGACACCATG-3'
	P	AAGGTCGGAGTCAACGGATTTGGTC-3'
CXCL17	F	5'-GCTGCCACTAATGCTGATGT-3'
	R	5'-GAGCCATCTCCTAGAAGCCT-3'
	P	5'-CTCTGGCGACCCCTGGATTCAG-3'
IL33	F	5'-AGACTTCTGGTTGCATGCC-3'
	R	5'-TCCAGGATCAGTCTTGCATTC-3'
	P	5'-CTGGTCTGGCAGTGGTTTTTCACAC-3'
